# miRNA mediated downregulation of cyclase-associated protein 1 (CAP1) is required for myoblast fusion

**DOI:** 10.3389/fcell.2022.899917

**Published:** 2022-09-30

**Authors:** Anurag Kumar Singh, Amrita Rai, Anja Weber, Guido Posern

**Affiliations:** ^1^ Institute for Physiological Chemistry, Medical Faculty, Martin Luther University Halle-Wittenberg, Halle (Saale), Germany; ^2^ Department of Internal Medicine I, University Hospital Halle, Martin Luther University Halle-Wittenberg, Halle (Saale), Germany; ^3^ Department of Structural Biochemistry, Max Planck Institute of Molecular Physiology, Dortmund, Germany

**Keywords:** CAP1, CAP2, microRNA, muscle differentiation, myogenesis, post-transcriptional regulation, myosin heavy chain

## Abstract

Myoblast fusion is essential for the formation, growth, and regeneration of skeletal muscle, but the molecular mechanisms that govern fusion and myofiber formation remain poorly understood. Past studies have shown an important role of the actin cytoskeleton and actin regulators in myoblast fusion. The Cyclase-Associated Proteins (CAP) 1 and 2 recently emerged as critical regulators of actin treadmilling in higher eukaryotes including mammals. Whilst the role of CAP2 in skeletal muscle development and function is well characterized, involvement of CAP1 in this process remains elusive. Here we report that CAP1, plays a critical role in cytoskeletal remodeling during myoblast fusion and formation of myotubes. *Cap1* mRNA and protein are expressed in both murine C2C12 and human LHCN-M2 myoblasts, but their abundance decreases during myogenic differentiation. Perturbing the temporally controlled expression of CAP1 by overexpression or CRISPR-Cas9 mediated knockout impaired actin rearrangement, myoblast alignment, expression of profusion molecules, differentiation into multinucleated myotubes, and myosin heavy chain expression. Endogenous *Cap1* expression is post-transcriptionally downregulated during differentiation by canonical myomiRs miR-1, miR-133, and miR-206, which have conserved binding sites at the 3′ UTR of the *Cap1* mRNA. Deletion of the endogenous 3′ UTR by CRISPR-Cas9 in C2C12 cells phenocopies overexpression of CAP1 by inhibiting myotube formation. Our findings implicates *Cap1* and its myomiR-mediated downregulation in the myoblast fusion process and the generation of skeletal muscle.

## Introduction

Skeletal muscle is the largest tissue in the body, accounting for ∼40% of human body mass. A fundamental step in the late differentiation process of muscle cells is the fusion of mononucleated myoblasts to multinucleated myofibers (Rochlin et al., 2010; Abmayr and Pavlath, 2012; Demonbreun et al., 2015). Similarly, in response to injury, myogenic progenitor cells within the adult musculature are activated and fuse to regenerate myofibers. Many studies have provided insights regarding the mechanisms and molecular components that mediate skeletal muscle myoblast fusion. These include characterization of the proteins mediating cell-cell adhesion and recognition of pathways that relay fusion signals from the cell surface to the cytoskeleton (Rochlin et al., 2010; Abmayr and Pavlath, 2012). In spite of these insightful studies, a complete understanding of the mechanisms governing the fusion of individual myoblasts into myotubes is lacking.

Dramatic reorganization of the cytoskeleton occurs as myoblasts maneuver through the morphological changes associated with cell-cell fusion to form multinucleated myotubes. These morphological changes include myoblast migration, elongation to a bipolar shape, membrane alignment and fusion (Rochlin et al., 2010; Demonbreun et al., 2015). In the live embryos, dynamic F-actin foci found at the point of cell-cell contact are formed and dissolves, which coincided with the myoblast fusion event (Kim et al., 2007; Richardson et al., 2007). In flies, a dense actin focus in fusion-competent myoblasts invades into founder cells through a thin sheath of F-actin, which requires the action of the nucleation-promoting factors (NPFs) Scar and WASP on the Arp2/3 complex (Sens et al., 2010; Haralalka et al., 2011). Similar to flies, extensive cytoskeletal reorganization occurs before and after fusion in cultured mammalian myoblasts. Visualization of the F-actin cytoskeleton revealed dynamic changes in fusing mammalian myoblasts *in vitro* and a dense F-actin wall paralleling the long axis of aligned myoblasts (Duan and Gallagher, 2009; Nowak et al., 2009; Stadler et al., 2010). As fusion proceeds, gaps appear in this actin wall at sites of vesicle accumulation, and the fusion pores form. In the absence of proper remodeling, F-actin structures continuously accumulate at the site of cell-cell contact and are correlated with a decrease in myoblast fusion (Nowak et al., 2009).

Cyclase-Associated Proteins (CAP) are evolutionarily conserved proteins with largely unknown physiological functions. Early studies suggested a rather passive role for CAP in actin cytoskeleton regulation, which was believed to act *via* sequestering globular actin monomers (Hubberstey and Mottillo, 2002). This view has changed considerably in the last decade because CAP has been implicated in almost all steps relevant for actin dynamics. Specifically, these studies pointed towards three major pathways how CAP protein regulate the actin treadmilling 1) a co-operation of CAP with key actin regulators such as ADF/Cofilin and Twinfilin in F-actin disassembly, 2) a nucleotide exchange activity on G-actin that is required for F-actin assembly, and 3) an inhibitory function towards the F-actin assembly factor inverted formin 2 (INF2) (Johnston et al., 2015; Kotila et al., 2019; Shekhar et al., 2019; Mu et al., 2020). Unlike yeast, mammals possess two Cap family members, *Cap1* and *Cap2*, with different expression patterns. CAP2 is abundant in the heart, striated muscle, and brain and is required for skeletal muscle development, heart physiology, and synaptic function (Peche et al., 2013; Field et al., 2015; Kepser et al., 2019; Pelucchi et al., 2020). Instead, CAP1 expression is less restricted, but its physiological functions remain largely unclear, due to the lack of an appropriate mouse model (Jang et al., 2020), while a recent brain-specific *Cap1* KO mouse study shows its role in the control of neuronal actin dynamics and growth cone morphology together with Cofilin1 (Schneider et al., 2021). Although embryonic expression in murine muscle has been observed for *Cap1*, it decreases postpartum to undetectable levels in the adult and has not been studied in relation to skeletal muscle development or differentiation (Bertling et al., 2004; Peche et al., 2007).

In this study, we report a downregulation in CAP1 expression during the differentiation of murine C2C12 and human LHCN-M2 cells from myoblast to myotubes. Loss- and gain-of-function experiments show that expression of CAP1 in myoblasts, as well as its differentiation-induced downregulation, is required for myotube formation. Further, we show that the downregulation of the *Cap1* expression is controlled *via* miRNA-mediated degradation of the *Cap1* mRNA, which results in the loss of CAP1 protein. We conclude that the timely loss of CAP1 is an important event during myoblast fusion into myotubes.

## Materials and methods

### Plasmids and reagents

Primers for qRT-PCR and sgRNA are listed in Supplementary Table S1. The cDNA clone for *Cap1* was purchased from Origene (CAT#: MR207594) and subsequently cloned into lentiviral vector pLVX-puro (Clontech, Mountain View, United States). For overexpression of the candidate miRNAs, mirVana mimics (Thermo Fisher) were used together with RNAi-max (Thermo Fisher) following the manufacturer’s instructions.

### Cell culture

C2C12 (DSMZ—German Collection of Microorganisms and Cell Cultures) was cultured subconfluently in Dulbecco’s modified Eagle’s medium (DMEM) supplemented with 10% fetal bovine serum (FBS), 2 mM L-glutamine, 1 mM sodium pyruvate, and antibiotic-antimycotic (Thermo Fisher) at 37°C and 5% CO_2_. Differentiation in C2C12 was induced by changing the medium to DMEM containing 2% horse serum (HS), 2 mM L-glutamine, 1 mM sodium pyruvate as well as antibiotic-antimycotic. Human immortalized LHCN-M2 myoblasts (Evercyte, Vienna, Austria; Cat. no. CkHT-040-231-2) were cultured in MyoUp medium (Evercyte, MHT-040). Following seeding, differentiation in the LHCN-M2 cells was induced by changing the medium (DMEM*/*M199 4:1, HEPES 20 mM, Zinc Sulfate 0.03 µg*/*ml, Vitamin B12 1.4 µg*/*ml, insulin 10 µg*/*ml and apotransferrin 100 µg*/*ml) and replacing it every second day.

For the generation of the *Cap1* knockout (KO) C2C12 cells, two guide RNAs were used (Supplementary Table S1). The guide RNAs were designed by using the tool CHOPCHOP (Labun et al., 2019) (https://chopchop.cbu.uib.no/) and were chosen based on the fact they did not show any off-target predictions. Pools of knockout cells were validated for the specificity of the *Cap1* deletion based on appearance of ∼1.3 kb PCR fragment corresponding to the double deletion in case of the knockout cells compared to the ∼7 kb fragment in the wild type cells, using Long Amp polymerase (M0323S; NEB). To generate the C2C12 cells carrying an endogenous deletion of the *Cap1* 3′-UTR, two guide RNAs (Supplementary Table S1) were designed to delete the majority of the 3′-UTR from the *Cap1* gene locus without affecting the coding region or the polyadenylation signal. sgRNA sequences were cloned into the lentiCRISPR-V2 vector (Addgene; Plasmid # 52961, a gift from Feng Zhang) (Sanjana et al., 2014). C2C12 cells were lentivirally infected and selected with puromycin for 1 week. CRISPR and control pools of C2C12 cells were characterized for genetic deletion of the 3′ UTR by PCR using primers flanking the two sgRNA using Taq DNA polymerase (M0267S; NEB). Furthermore, the smaller PCR fragments were cloned and sequenced for the validation of the deleted *Cap1* 3′ UTR. Generation of monoclonal cell lines is not possible because myoblasts have to be cultured subconfluently to maintain their undifferentiated state and fusion potential.

### Reverse transcription-quantitative PCR

RNA was extracted using NucleoSpin RNA Mini kit for RNA purification (Macherey-Nagel Inc.) and cDNA was synthesized using Verso cDNA Synthesis Kit (Thermo Fisher Scientific), following the manufacturer’s instructions. Real-time PCR amplification and analysis were performed using a LightCycler 480 with Dynamo ColorFlash SYBR Green (Roche) and the primers are listed in Supplementary Table S1. For normalization, two different housekeeping mRNAs were used as controls. Calculations were done using the ΔΔ cycle threshold (Ct) method (Pfaffl, 2001). For statistical analysis, the Student’s t-test was applied.

### Immunoblotting

Western blot analysis was performed following standard protocols. Primary antibodies against CAP1 (sc-376286; dilution: 1:500, Santa Cruz), Tubulin (T9026; 1:5,000, Sigma), Flag (F7425; 1:1,000, Sigma), Myosin Heavy chain 1*/*2*/*4*/*6 (sc-32732; 1:1,000, Santa Cruz), GAPDH (5174S; 1:3,000, Cell Signaling), Myogenin (sc-12732; 1:1,000) and MyoD (MA1-41017; 1:1,000, Thermo Fisher Scientific) were incubated overnight at 4°C. Fluorophore-conjugated secondary antibodies IRDye 700 or IRDye 800 (1:15,000, LICOR Biosciences) were incubated for 1 h at room temperature. Imaging and quantifications were done using the Odyssey Image Scanner System with the software Image Studio V 3.1.4 (LI-COR Biosciences, Cambridge, United Kingdom), as described before (Werner et al., 2019).

### F-actin and crystal violet staining (cell size measurement)

For F-actin staining, cells were seeded in 12 well plates for 24 h, followed by differentiation as indicated. Cells were fixed with 3.7% PFA for 15 min and permeabilized with 0.1% Triton X-100 for 10 min. After washing the cells were incubated with phalloidin (Alexa Fluor™ 488 phalloidin, Thermo Scientific) in the blocking buffer following the manufacturer’s instruction. For crystal violet staining and cell size measurements, cells were seeded in a similar fashion at a sub-confluent level in 12 well, plate, and post 24 h they were incubated with 0.5% crystal violet in methanol for 20 min. The cell size measurement was done by ImageJ software.

### Immunofluorescence microscopy for myosin heavy chain

For immunofluorescence microscopy cells were fixed with 3.7% formaldehyde, permeabilized with 0.2% Triton X-100 and blocked with 10% horse serum (Sigma Aldrich, München, Germany), 1% BSA (Carl Roth), 0.05% Triton X-100 (Carl Roth) in PBS. The following antibodies were used: Myosin Heavy chain 1*/*2*/*4*/*6 (1:500, Santa Cruz) and secondary antibodies conjugated with Alexa488 (1:500, Thermo Fisher Scientific). DNA was counterstained with DAPI (Sigma Aldrich). Samples were covered with Immu-Mount (Thermo Fisher Scientific) and imaged with an EVOS fl Fluorescence Microscope (AMG, Thermo Fisher).

### Transient transfection of the miRNA mimics

Both the C2C12 and LHCN-M2 cells were transiently transfected with miRNA mimics (mirVana mimics, Thermo Fisher). Briefly, the cells were seeded at sub-confluent density in the six well plate format and were transfected with 50 nmoles of miR-Ctl, miR-1a-3p, miR-133a-3p, miR-206-3p, miR-378-3p, or miR-486-5p mimics using 10 µl of RNAimax following the manufacturer’s instruction. After 72 h, cells were lysed and analyzed for the effect of the miRNA transfection on *Cap1* mRNA and protein level by reverse transcription quantitative PCR and immunoblotting respectively.

## Results

### *Cap1* mRNA and protein levels are downregulated during myogenic differentiation

**FIGURE 1:**
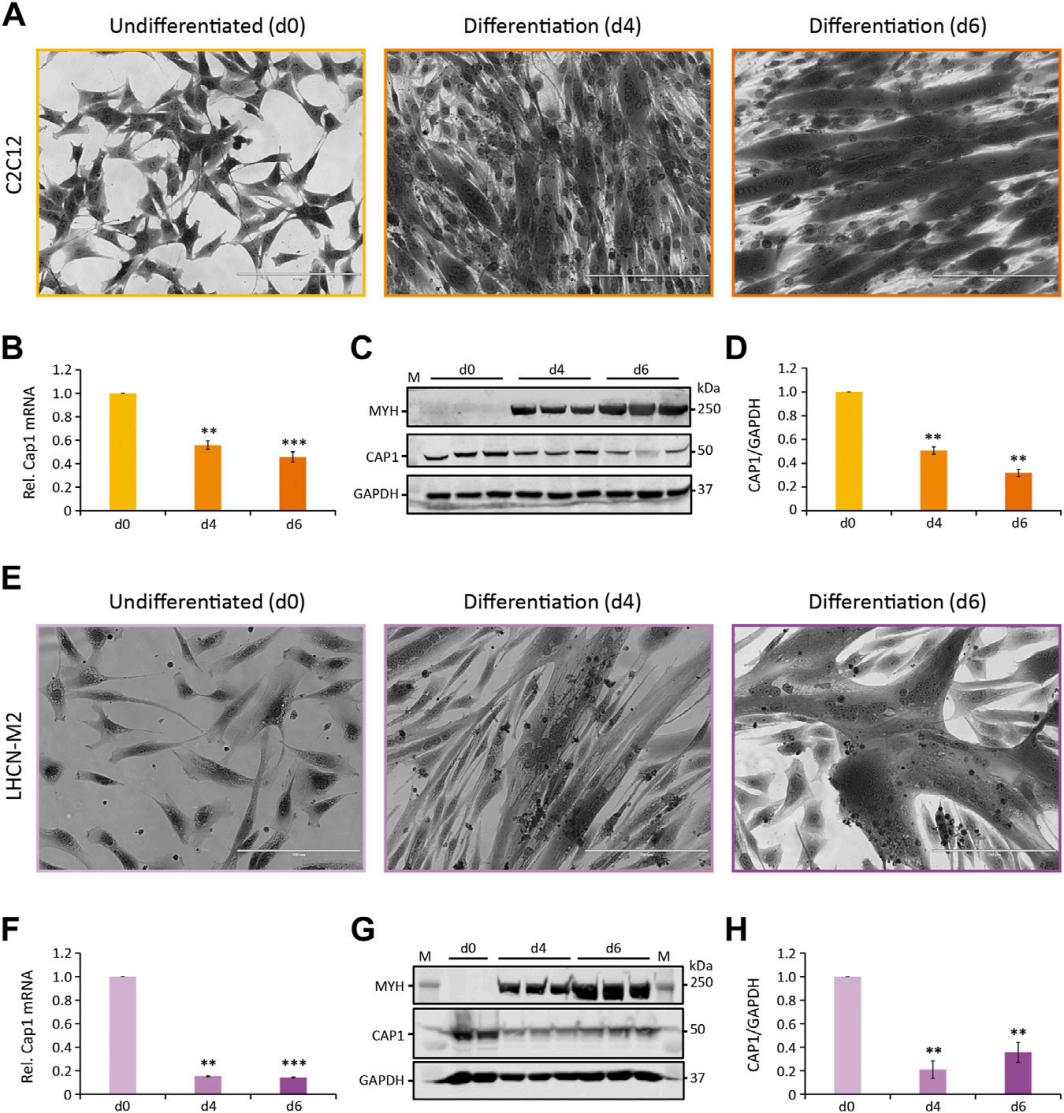
*Cap1* mRNA and protein levels are downregulated during myogenic differentiation. **(A,E)** Bright-field images (×20) of murine C2C12 **(A)** and human LHCN-M2 **(E)** cells upon differentiation for 4 and 6 days (d4 and d6), in comparison to undifferentiated control cells (d0). Cells were stained with crystal violet. **(B,F)** Relative *Cap1* mRNA in differentiating C2C12 **(B)** and LHCN-M2 **(F)** cells, quantified by qRT-PCR and normalized to a set of housekeeping mRNAs. **(C,G)** Immunoblots of lysates from differentiating C2C12 **(C)** and LHCN-M2 **(G)** cells, using antibodies against myosin heavy chain polypeptides 1, 2, 4, and 6 (MYH), CAP1 and GAPDH as a control. **(D,H)** Quantification of CAP1 immunoblots at myogenic differentiation for 4 and 6 days, normalized to undifferentiated control cells. *Error bars*, SEM (*n* = 3); **p < 0.01, ***p < 0.001 (Student’s *t*-test). Scale bar, 200 μm.

Both murine and human skeletal muscle cell lines C2C12 and LHCN-M2 are able to recapitulate terminal differentiation of myoblasts to fused, multinuclear myotubes. To elucidate this process, cells were seeded at high density, and differentiation was induced by changing to a differentiation medium. The first multinucleated myotubes were observable at day 4 (Figures 1A,E, d4) with more mature myotubes at day 6 (Figures 1A,E, d6). Subsequently, the differentiation process was verified by the appearance and subsequent upregulation of myosin heavy chain polypeptides MYH1, MYH2, MYH4, and MYH6 over the course of differentiation (Figures 1C,G).

CAP proteins have been implicated in the regulation of actin treadmilling and the differentiation of muscle cells. Because of the importance of actin remodeling during myoblast fusion, we analyzed whether or not CAP1 plays any role in myogenic differentiation. Muscle expression of *Cap1* at the embryonic stage had been documented, but it decreases postpartum and is undetectable in adult mice (Bertling et al., 2004). We thus assessed *Cap1* expression during C2C12 and LHCN-M2 differentiation both at mRNA as well as protein level. *Cap1* mRNA and protein were clearly expressed in undifferentiated cells but decreased upon differentiation (Figures 1B–D,F–H). In contrast, *Cap2* mRNA was hardly detectable in undifferentiated myoblasts but was upregulated in differentiating cells, consistent with its previously described role in postnatal skeletal muscle development (Supplementary Figure S1) (Kepser et al., 2019).

To further validate the inverse regulation of *Cap1* and *Cap2* during myogenic differentiation and regeneration, we re-analyzed two existing microarray datasets from the GEO database (Edgar et al., 2002). The first one investigated the genome-wide mRNA expression of C2C12 cells differentiating from myoblasts to myotubes (GSE4694) (Chen et al., 2006). The second one analyzed the gene expression in the mouse *Tibialis* anterior (TA) muscle of 12 week old C57BL/6J males after injury by glycerol injection for up to 21 days (GSE45577) (Lukjanenko et al., 2013). The data analysis revealed a significant upregulation of *Cap1* immediately post TA injury, with subsequent downregulation towards 21 days as the injury heals and the muscle is regenerated *in vivo* (Supplementary Figure S2). Consistent with this and our data, *Cap1* is also expressed in the dataset of C2C12 myoblasts, but downregulated during myotube formation *in vitro* (Supplementary Figure S3) (Chen et al., 2006). In contrast, both studies find increased levels of *Cap2* upon differentiation *in vitro* or regeneration *in vivo*, in line with our observation of inversely regulated *Cap1* and *Cap2* mRNA in differentiating C2C12 or LHCN-M2 cells. These data demonstrate CAP1 expression in myoblasts and suggest a role for timely downregulation upon differentiation.

### *Cap1* deletion results in increased cell size and F-actin organization

**FIGURE 2:**
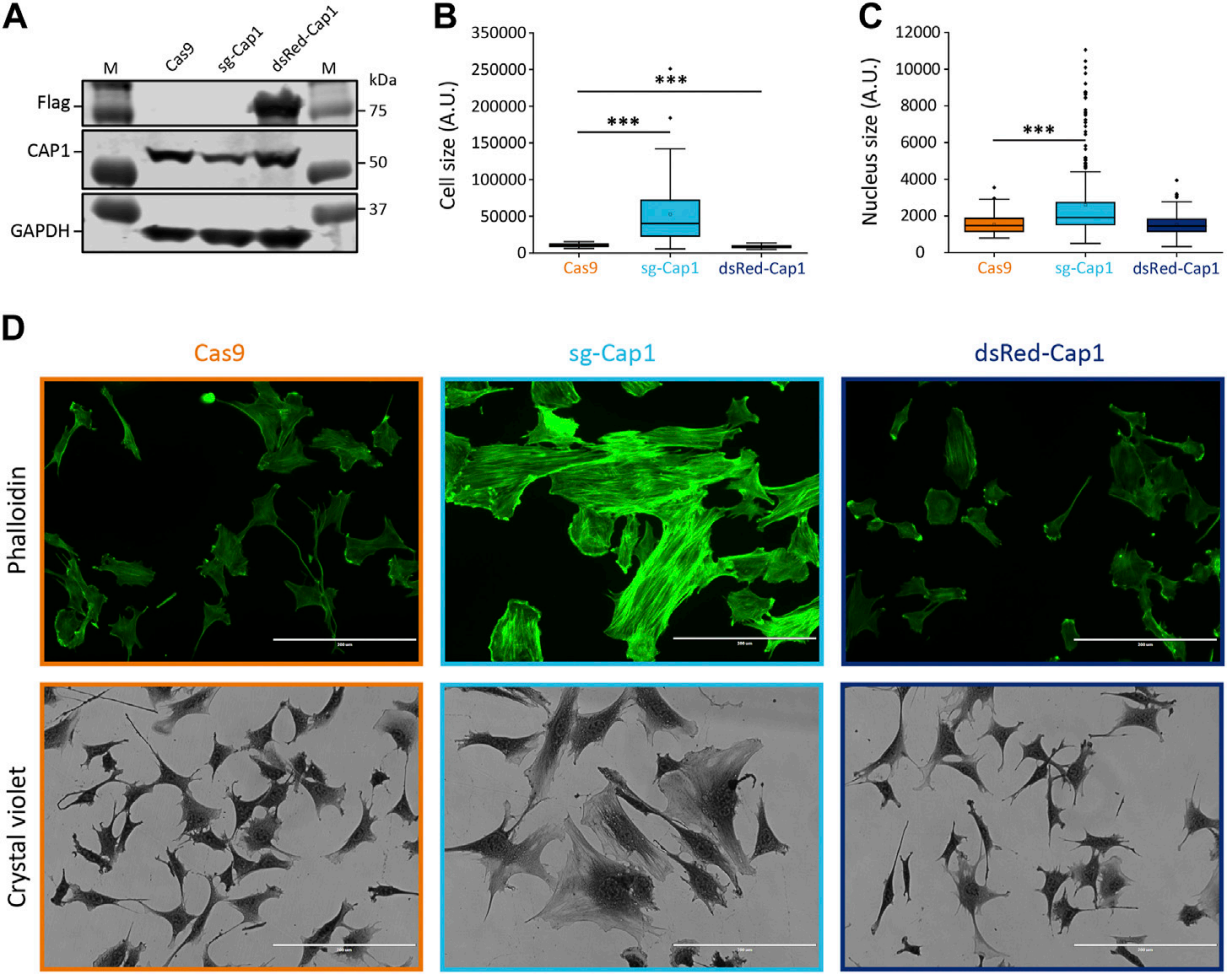
Knockout of *Cap1* results in increased size of cells and nuclei and accumulated F-actin fibers in C2C12 cells. **(A)** Validation of partial knockout by CRISPR-Cas9 (sg-Cap1) and overexpression (dsRed-Cap1) in C2C12 pools by immunoblot, compared to control cells infected with Cas9 only (Cas9). The sg-Cap1 cells show reduced expression of endogenous CAP1 while ectopically expressed dsRed-Cap1 results in an additional band corresponding to tagged CAP1 proteins (*n* = 3). **(B)** Quantification of the cell area covered, based on crystal violet staining (*n* = 50 cells). **(C)** Quantification of the nucleus size (*n* = 100 nucleus). **(D)** Micrographs, the upper panel shows fluorescence images of phalloidin-stained cells. The lower panel shows bright-field images (×20) of crystal violet stained cells. Data presented here are from two-week post transduction. The cell and nucleus size quantifications are presented as box plot, showing mean (cross), median (line), 25th and 75th percentile (box). The whiskers extend to the most extreme data points not considered outliers, and the outliers are represented as dots; ***p < 0.001 (Student’s *t*-test). Scale bar, 200 μm.

To investigate whether the observed decrease in *Cap1* expression plays a role in myogenic differentiation, we perturbed the expression of CAP1 and assessed if that affects the myoblast fusion and myogenic differentiation. First, we analyzed whether or not the knockout (KO) of *Cap1* has any impact on F-actin. Using CRISPR-Cas9 and two sgRNA, we selected a pool of cells harboring the intended deletion at the *Cap1* gene locus (Supplementary Figure S4). The control (Cas9) cells shows 7 Kb amplicon corresponding to the size of the primer pairs flanking the two guide-RNA, while, the knockout cells shows an additional smaller fragment (∼1.3 Kb) corresponding to the double deletion. The 7 Kb fragment in the knockout pools might also indicate single or double deletions with small in/del mutation. In line with our PCR analyses, the partial knockout showed reduced CAP1 protein abundance (Figure 2A). This resulted in increased size of the cells and nuclei, a spread-out morphology and enhanced F-actin filament formation, resulting in stable stress fiber (Figures 2B–D; Supplementary Figure S5). This result is consistent with the role of CAP1 as an F-actin depolymerizing protein (Kotila et al., 2019; Shekhar et al., 2019). In contrast, cells overexpressing CAP1 (Flag tagged dsRed-Cap1) looked significantly smaller as compared to the undifferentiated Cas9 control cells, with no difference in the nuclear size or F-actin staining (Figures 2B–D; Supplementary Figure S5).

### Deregulated *Cap1* expression inversely correlates with myogenic differentiation

**FIGURE 3:**
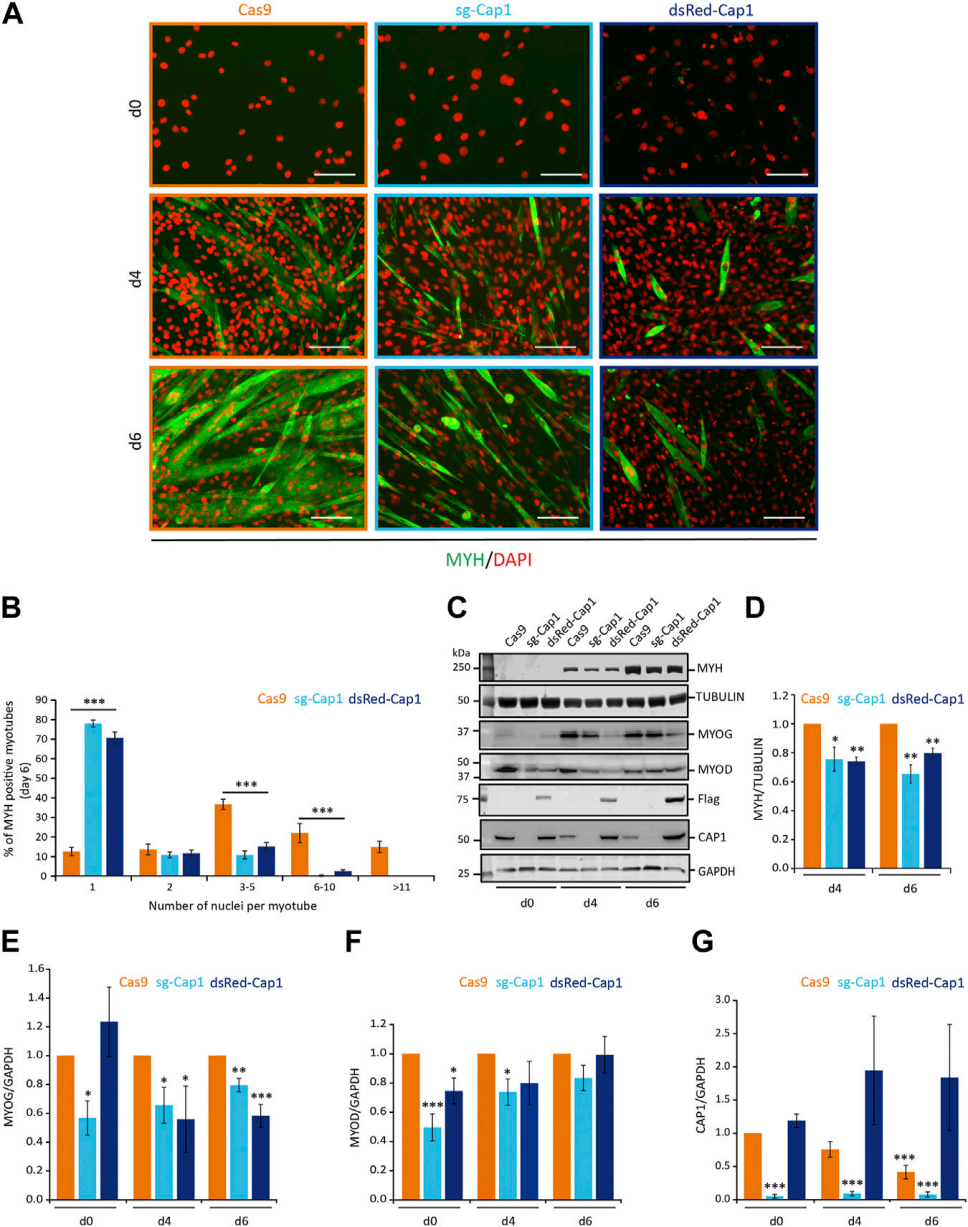
Timely downregulation of CAP1 is important for myoblast fusion. **(A)** Cas9 control cells, knockout (sg-Cap1) and overexpressing (dsRed-Cap1) cells at day 0 (d0), day 4 (d4) and day 6 (d6) of their differentiation. Pools of C2C12 cells were stained for MYH (green) and nucleus (DAPI, orange). **(B)** Quantification of the percentage of myotubes containing the indicated number of nuclei per myotube in control, knockout and overexpressing myotubes after 6 days of differentiation (minimum 200 MYH positive myotubes were counted). **(C)** Western blot for the myogenic marker MYH, TUBULIN, MYOG, MYOD, Flag, CAP1, and GAPDH at day 0 (d0), days 4 (d4) and 6 (d6) of the differentiation. **(D–G)** Quantification of MYH, MYOG, MYOD, and CAP1 immunoblots at myogenic differentiation for 4 and 6 days, normalized to Cas9 control cells. *Error bars*, SEM (*n* = 3); *p < 0.05, **p < 0.01, ***p < 0.001 (Student’s *t*-test). Scale bar, 200 μm.

F-actin reorganization is critical for myoblast fusion and myogenic differentiation. To test whether CAP1 downregulation marks an important event, we studied the differentiation process of the C2C12 cells with perturbed expression of CAP1. Under both circumstances (*Cap1* overexpression and knock-out), the differentiation was severely affected (Figure 3; Supplementary Figure S6). On day 2, the control cells were spindle-shaped and aligned to each other (Supplementary Figure S6, Cas9). The *Cap1* KO cells seemed bigger and more elongated, while the CAP1 overexpressing cells lacked this phenotype at day 2 and maintained a fibroblast-like phenotype, similar to undifferentiated cells (Supplementary Figure S6, d2). Both the overexpression and the knockout of the *Cap1* changed the way cells align to each other at the initial phase of differentiation and affect the fusion process. The result suggests an important role for the timely downregulation of the CAP1 protein during the differentiation process. In accordance, cell movement and change in cell shape followed by cell-cell adhesion are known to be important steps during myoblast fusion and myogenic differentiation.

Further, we analyzed the expression of the differentiation marker myosin heavy chain (MYH) by immunofluorescence microscopy (Figure 3A) and western blot analysis (Figures 3C,D). Compared to the Cas9 control cells, the MYH positive myotubes appeared thinner and with less number of nuclei per myotube upon *Cap1* knockout and overexpression (Figures 3A,B). Western blot analysis confirmed that both overexpression and *Cap1* knockout resulted in decreased levels of MYH over the course of differentiation (Figures 3C,D). Next, we analyzed the number of nuclei present in the MYH-positive myotubes at day 6 of differentiation. Cas9 control myotubes showed significantly higher numbers of nuclei compared to both the knockout as well as CAP1 overexpressing cells (Figure 3B). In contrast, the majority of MYH-positive “myotubes” with altered CAP1 expression contained only one nucleus.

We also checked for the expression of the two myogenic transcription factors Myogenin (MYOG) and Myoblast Determination Protein (MYOD). The knockout cells showed a significant decrease in both the tested transcription factors at undifferentiated state as well as at day 4 of the differentiation (Figures 3C,E,F). At day 6, the expression of MYOD was not different as compared to the Cas9 control cells, while Myogenin was significantly downregulated. CAP1 overexpression also resulted in significant downregulation of MYOD at undifferentiated state and at day 4 and day 6 for the Myogenin (Figures 3C,E,F).

As mentioned before the control cells showed a constant decrease in the expression of the CAP1 protein level over the course of differentiation. The knockout cells (sg-Cap1) barely showed any expression of the CAP1 at any stage of the differentiation (Figures 3C,G). In contrast, CAP1 overexpressing cells did not show any decrease in the CAP1 expression during differentiation (Figures 3C,G). Overexpression of CAP1 was validated by anti-Flag antibody since the CAP1 antibody failed to detect the overexpressed CAP1 fused to an N-terminal dsRed or harboring an N-terminal deletion (Cap1-Ct; Supplementary Figure S7).

These results point towards a defect in the fusion process during the differentiation. Since the expression of CAP1 was decreasing upon differentiation (Figures 1, 3C,G), and perturbation of CAP1 expression impaired differentiation, we conclude that a timely decrease of CAP1 is paramount to myoblast fusion and maturation.

### *Cap1* knockout, as well as overexpression, resulted in aberrant pre-fusion F-actin organization

**FIGURE 4:**
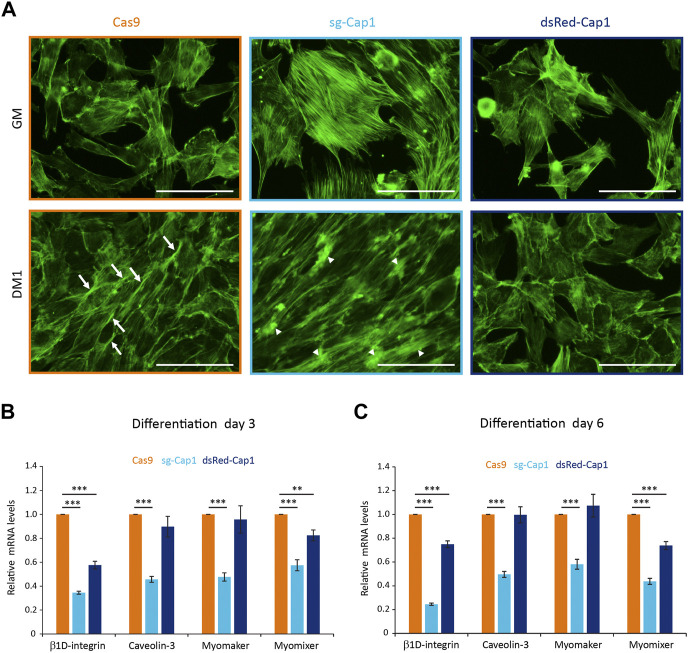
Disturbance of the early cortical actin rearrangement and expression of pro-fusion molecules upon changes in CAP1 expression. **(A)** Cas9 control, sg-Cap1 and dsRed-Cap1 cells were cultured in growth medium (GM) or differentiation medium (DM1; day 1 of differentiation) and fixed and stained to detect F-actin using phalloidin. Aligned Cas9 control cells (lower panel) show longitudinal actin fiber accumulation at sites of contact (arrows), whereas *Cap1* knockout cells (sg-Cap1, middle-lower panel) exhibit mislocalized, thickened actin patches (arrowheads). Alignment and cortical actin fibers are absent in dsRed-Cap1 cells (last panel). Scale bar, 100 μm. **(B,C)** The mRNAs for *ß1D-integrin*, *Caveolin-3*, *Myomaker*, and *Myomixer* were quantified by qRT-PCR and normalized to a set of housekeeping mRNAs at day 3 **(B)** and day 6 **(C)** of differentiation. *Error bars*, SEM (*n* = 3); **p < 0.01, ***p < 0.001 (Student’s *t*-test).

During differentiation of myoblast to myotubes, the actin filaments undergo dramatic reorganization. In the mammalian system, it has been shown that myoblasts develop a non-uniform thickened actin wall as a prerequisite for the fusion event (Duan and Gallagher, 2009). Thus, we looked for changes in the F-actin organization after 1 day in the differentiation medium and whether it was affected by altering the expression of CAP1 in C2C12 cells. Compared to Cas9 control cells which show F-actin bundles at the lateral sides of the aligned spindle-shaped cells (Figure 4A, arrows), *Cap1* knockout cells (sg-Cap1) showed the frequent appearance of F-actin foci and thick disorganized accumulation of F-actin (Figure 4A, arrowheads). In contrast, CAP1 overexpressing cells (dsRed-Cap1) lacked the elongated cell phenotype and the F-actin organization appeared similar to the undifferentiated cells, consistent with a delayed response to the differentiation medium.

### Perturbation in CAP1 expression resulted in the downregulation of pro-fusion molecules

Myoblast fusion requires several cell adhesion and transmembrane proteins, which accumulate at the contact sites between two myogenic cells. To further understand the role of *Cap1*, knockout and CAP1 overexpressing cells were incubated in the differentiation medium and relative mRNA levels of the profusion molecules *ß1D-integrin*, *Caveolin-3*, *Myomaker*, and *Myomixer* were quantified by qRT-PCR. Interestingly, all profusion mRNA were significantly reduced in the *Cap1* KO cells compared to the Cas9 control (Figures 4B,C). In the case of CAP1 overexpressing cells, only the levels of *ß1D-integrin* and *Myomixer* decreased significantly (Figures 4B,C). This indicates that the expression of profusion molecules is more severely affected by *Cap1* knockout than by its overexpression, although the final result of myotube formation is impaired in both cases. Together, the results suggest that CAP1 perturbations impair differentiation by distinct mechanisms: whereas depletion hampers fusion, continuous overexpression of CAP1 affects myoblast elongation and alignment at the initial stage of differentiation.

### Regulation of *Cap1* expression by known myomiRs during differentiation

**FIGURE 5:**
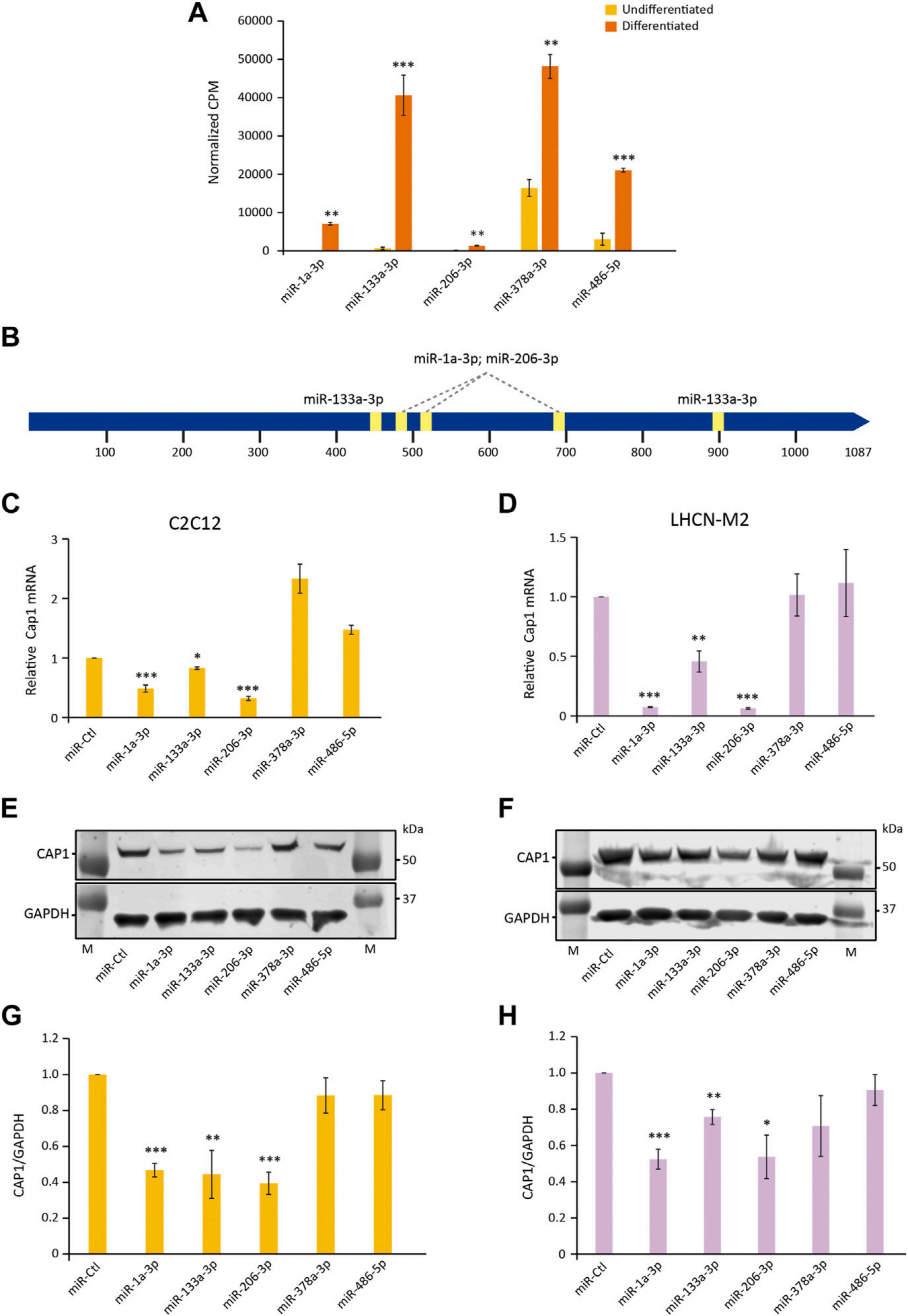
miRNA (miR-1, miR-133, and miR-206) regulate the expression of *Cap1* in murine and human myoblast. **(A)** The abundance of the indicated miRNAs in total lysates of undifferentiated and differentiated C2C12, determined by RNA-Seq. CPM; counts per million (*n* = 3). **(B)** Schematic of the 3′-UTR of murine *Cap1* with the STOP-codon at position 1 and the polyadenylation signal at 1,020 and 1,058 bp. Predicted binding sites for miR-1, miR-133 and miR-206 are indicated by yellow boxes. **(C,D)** *Cap1* mRNA expression in undifferentiated C2C12 **(C)** and LHCN-M2 **(D)** cells transfected with the indicated miRNA mimic for 72 h (*n* = 3). **(E,F)** Representative immunoblots of cells transfected with the indicated miRNA. **(G,H)** Quantification of the CAP1 protein from three independent experiments. Error bars, SEM (*n* = 3); *p < 0.05, **p < 0.01, ***p < 0.001 (Student’s *t*-test).

Since *Cap1* is downregulated both at the mRNA and the protein level, we looked for the mechanism that might regulate the expression of *Cap1* during differentiation. It is well established that the differentiation process of skeletal muscle involves miRNA called myomiRs which are upregulated manifold during differentiation (Sjogren et al., 2017; Holstein et al., 2020; Singh et al., 2020; Xu et al., 2020). We thus revisited our own published dataset (GSE136956) (Holstein et al., 2020) for differentiation-induced miRNA which might have potential binding sites at the 3′-UTR of *Cap1*. Based on predicted binding sites at the *Cap1* mRNA we choose miR-1, miR-133, and miR-206, whereas miR-378 and miR-486 served as controls (no predicted binding site at the 3′-UTR of *Cap1* mRNA) (Figures 5A,B; Supplementary Figure S8).

Upon transfection of the corresponding miRNA mimics, *Cap1* expression is downregulated by miR-1, miR-133 and miR-206 both at mRNA and protein level in C2C12 and LHCN-M2 cells (Figures 5C–H). In contrast, miRNAs which are shown to be upregulated during differentiation but lack predicted binding sites at the 3′ UTR did not downregulate the expression of the *Cap1*. The result suggests a posttranscriptional regulation of *Cap1* expression by specific miRNAs, involved in the myogenic differentiation process.

### *Cap1* reduction and myogenic differentiation requires post-transcriptional regulation *via* its 3′-UTR

To investigate whether *Cap1* expression is directly regulated at the mRNA level, the 3′-UTR of the endogenous *Cap1* locus was deleted by CRISPR–Cas9, using sgRNAs flanking the putative miRNA binding sites. Pools of infected and selected C2C12 cells were validated by PCR and sequencing of several cloned genomic loci, showing that the targeted region of the 3′-UTR between the STOP and the polyadenylation signal was successfully deleted (Supplementary Figure S9).

**FIGURE 6:**
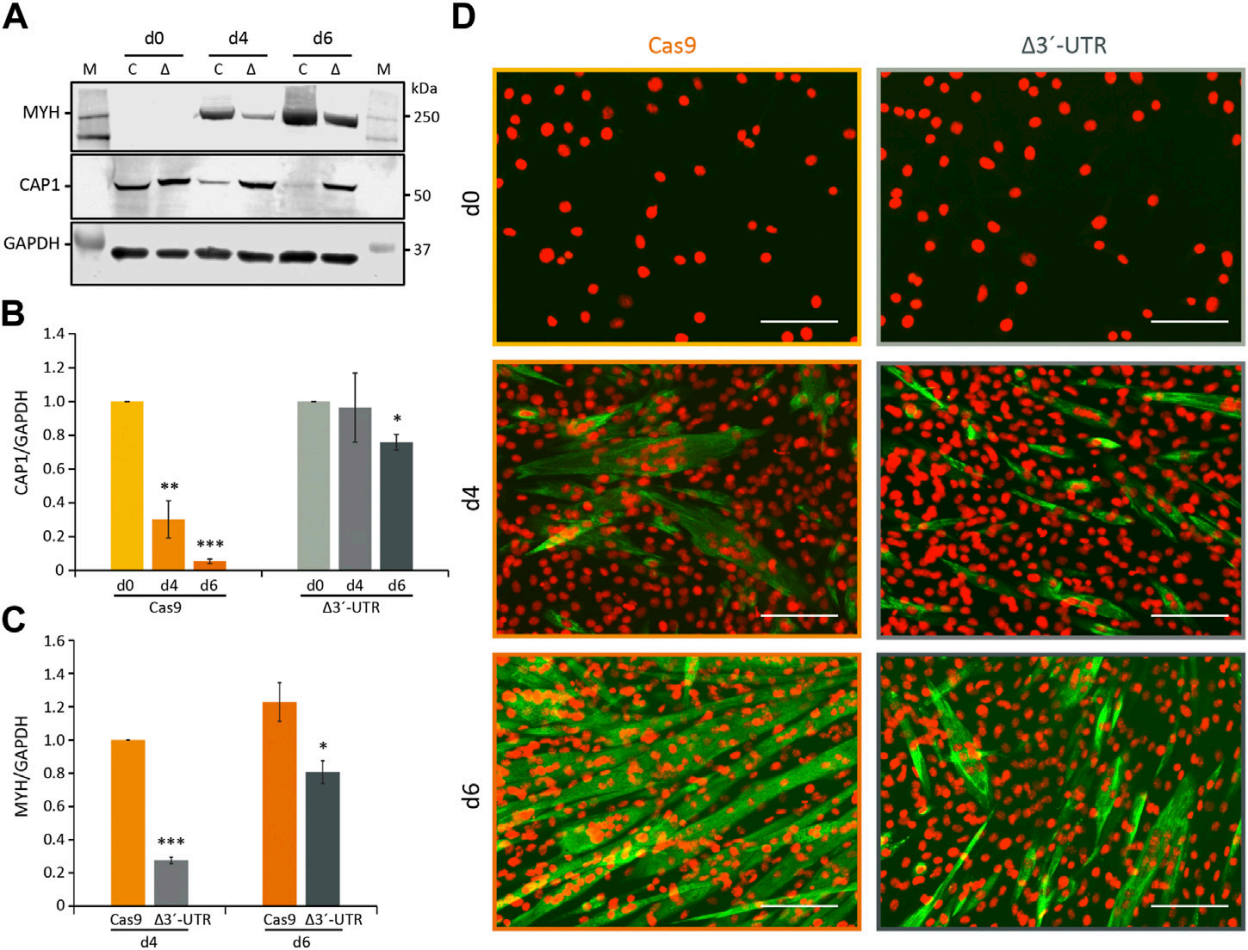
Requirement of the 3′-UTR for *Cap1* regulation during myogenesis. **(A)** The 3′-UTR of *Cap1* was deleted in C2C12 cells using CRISPR/Cas9. Δ3′-UTR (Δ) and Cas9-only (C) control cells were differentiated for the indicated times. Representative immunoblots for MYH and CAP1 are shown. **(B)** Quantification of CAP1 protein at day 0, day 4 and day 6 of differentiation normalized to day 0. **(C)** Quantification of MYH, normalized to Cas9 control cells at day 4. **(D)** Immunofluorescence staining (×20) of C2C12 cells stained with MYH (green) and DAPI (orange) after differentiation for 4 and 6 days (d4 and d6), in comparison to undifferentiated control cells (d0). Thick and multinucleated myotubes are reduced in the Δ3′-UTR cells (right panel) at day 6 of differentiation, compared to the Cas9 control (left panel). Error bars, SEM (*n* = 3); *p < 0.05, **p < 0.01, ***p < 0.001 (Student’s *t*-test). Scale bar, 200 μm.

These ΔUTR C2C12 cells showed no reduction of CAP1 protein during differentiation, compared to the Cas9 control cells, which exhibited the significantly decreasing CAP1 expression observed before (Figures 6A,B). In addition, myotube formation was impaired in cells with deleted *Cap1* 3′-UTR. The MYH marker of myogenic differentiation was reduced in the 3′-UTR-deleted cells, as shown for 4 and 6 days post differentiation (Figures 6A,C,D). Morphologically, the ΔUTR C2C12 cells showed less mature, less multinucleated myotubes, compared to the Cas9 control cells (Figure 6D). This appears to be reminiscent of the CAP1 overexpressing cells (Figure 3; Supplementary Figure S6). Together, these results indicate that posttranscriptional downregulation of *Cap1 via* its 3′-UTR is critical for myogenic differentiation, and suggest that a timely downregulation of *Cap1* is important for controlling F-actin mediated changes of cell shape during maturation.

## Discussion

In the present study, we show a critical role of both CAP1 expression and its subsequent downregulation during myogenic differentiation. Our report implicates the miRNA-mediated downregulation of *Cap1* in the myoblast-to-myotube differentiation. Myogenesis-induced upregulation of the myomiRs miR-1, miR-133 and miR-206 inhibit *Cap1* expression, which in turn reduces the CAP1 protein amount. This enables the reorganization of F-actin that is required for changes in myoblast morphology and eventually the fusion process.

*Cap1* is expressed in many tissues, but its physiological function remains largely unclear. *Cap1* knockout mice do not survive post-embryonic day 16.5 (Jang et al., 2020). A recent brain-specific *Cap1* knockout shows a role in neuronal actin dynamics and growth cone morphology together with Cofilin1 (Schneider et al., 2021). *Cap1* in relation to skeletal muscle development or differentiation was not yet studied, probably due to almost undetectable levels of CAP1 in the adult skeletal muscle (Bertling et al., 2004; Peche et al., 2007). However, skeletal muscle *Cap1* expression is detectable at the embryonic state, which reduces dramatically at birth and almost completely disappears in the adult. Similarly, *Cap1* expression was found in microarray analysis of the skeletal muscle injury model, where *Cap1* expression is upregulated immediately post-injury and disappears as the injury heals (Lukjanenko et al., 2013) (Supplementary Figure S2). Together with our data presented here, supports a role for CAP1 in muscle development and/or differentiation.

Both murine C2C12 and human LHCN-M2 are immortalized myoblast cells that partially recapitulate the myogenic differentiation *in vitro*. Both cell lines express CAP1, followed by a similar decrease upon induction of differentiation (Figure 1), in agreement with microarray expression analysis previously performed by others (Chen et al., 2006) (Supplementary Figure S3). Thus, CAP1 might be expressed in activated skeletal muscle stem cell populations and myoblasts, while its expression decreases with maturation. In contrast, CAP2 is abundant in adult muscle and myotubes, and it is required for heart function and postnatal skeletal muscle development in mice and humans (Peche et al., 2013; Field et al., 2015; Stöckigt et al., 2016; Aspit et al., 2019; Kepser et al., 2019; Schneider et al., 2021). Due to its abundant expression, most investigations of CAP proteins in muscle have thus focused on *Cap2*, but no roles in the early steps of myogenic differentiation have yet been characterized.

To date, a variety of experimental approaches have provided a basic framework of the processes that are required for myoblast fusion. On the cellular level, mammalian myoblasts seem to have an initial fibroblast-like morphology, which subsequently develops into an elongated, spindle-like shape capable of migrating towards other differentiating myoblasts (Ohtake et al., 2006; Nowak et al., 2009). Following contact, and membrane alignment, adhesion occurs between neighboring cells prior to membrane breakdown and fusion (Swailes et al., 2004; Ohtake et al., 2006; Peckham, 2008). During these early steps of differentiation, actin cytoskeleton rearrangements play a critical role. In a conditional Rac1 knockout mouse model, the recruitment of Arp2/3 and F-actin to the cell contact site is reduced, resulting in impaired myoblast migration and fusion (Vasyutina et al., 2009). Experiments using C2C12 cells demonstrated that WAVE-dependent actin remodeling is required during myoblast fusion (Nowak et al., 2009). In rat L6 myoblasts, the formation of a non-uniform cortical actin wall has been proposed, which provides structural and mechanical support to facilitate membrane alignment and fusion pore formation (Duan and Gallagher, 2009).

Concerning actin regulation, studies from the past decade have put CAP proteins at the center stage of the actin treadmilling (Ono, 2013; Kotila et al., 2019; Shekhar et al., 2019; Rust et al., 2020). Towards this end, we could show that CAP1 reduction impaired the F-actin wall formation between C2C12 cells upon induction of the differentiation process (Figure 4A). Rather, *Cap1* knockout resulted in dramatically increased F-actin accumulations at uncoordinated places. On the contrary, overexpression of the CAP1 led to a delayed response and cells still had a fibroblastoid shape, like the undifferentiated cell population (Figure 4A). Together these data suggest that a timely upregulation in the F-actin content and its correctly localized arrangement upon induction of the differentiation plays a crucial role in the early steps of myoblast fusion.

After induction of differentiation, myoblasts exhibit an increased F/G-actin ratio, while the F-actin content of the cells decreases again at later stages and the ratio comes back to normal (Duan and Gallagher, 2009). This early increase in F-actin could be explained by the timely downregulation of CAP1, since the experimental reduction of CAP1 results in increased F-actin (Figure 2) (Bertling et al., 2004; Zhang et al., 2013). Further, an increased expression of *Cap2* is observed during myogenic differentiation or regeneration, which potentially explains the normalization of the F/G-actin ratio towards completion of the process (Supplementary Figures S1–S3) (Chen et al., 2006; Lukjanenko et al., 2013). Indeed, *Cap2* upregulation during myogenesis is not essential for myoblast fusion or myogenic differentiation at the early stage, as evident from normal muscle development in *Cap2* knockout mice, but for the late α-actin exchange during skeletal myofibril differentiation (Kepser et al., 2019). Such a delay in the α-actin exchange has also been reported for mutant mice lacking Cofilin2, suggesting that CAP2 and Cofilin2 cooperate in myofibril differentiation (Gurniak et al., 2014). We speculate that CAP1 mediates actin regulation in the stem cell/myoblast compartment of the skeletal muscle and its downregulation increases appropriate F-actin structures needed for myoblast fusion, while the subsequent increase in CAP2 is needed to cut back the F-actin level and to play other important roles in myotube maturation.

Both overexpression as well as partial genetic knockout of CAP1 prevented proper differentiation of the C2C12 cells, as evident by a decrease in the signal for MYH in the western blot analysis (Figures 3C,D). Interestingly, perturbation of CAP1 expression most prominently decreased myoblast fusion, as evident by the thickness and the number of nuclei per myotube (Figures 3A,B). These results suggest that changes in the actin cytoskeleton by perturbing CAP1 expression primarily affect the fusion process rather than activation of differentiation programming as evident by the expression of MYH. This is in agreement with the previous result that shows a pharmacological inhibition of actin remodeling by Cytochalasin D or Latrunculin B did not affect the expression of differentiation marker MYH, while hampered the fusion of myoblasts (Nowak et al., 2009). Although differentiation is similarly impaired, the excessive F-actin might be causative in the *Cap1* knockout, while the delayed elongation may underlie the defects in CAP1 overexpressing cells (Figure 4A). Such distinct reasons for a common outcome are similarly described for other actin modulator: Loss of Diaphanous results in less F-actin formation and reduced myoblast fusion, while its gain-of-function mutant massively increased F-actin foci, but ultimately also prevented myoblast fusion (Deng et al., 2015).

Our results show that CAP1 can regulate the expression of the myoblast profusion molecules *ß1D-integrin*, *Caveolin-3*, *Myomaker*, and *Myomixer* (Figures 4B,C). In the past, it has been well established that the myogenic transcription factors MYOD and MYOG regulate the expression of *Caveolin-3*, *Myomaker*, and *Myomixer* (Biederer et al., 2000; Luo et al., 2015; Zhang et al., 2020). Although a direct regulation of *ß1D-integrin* has not been reported, it might be indirectly regulated by these transcription factors as they regulate the expression of its binding partners (Huijbregts et al., 2001; Luo et al., 2021). Since both the knockout as well as the overexpression of the CAP1 resulted in similar decrease in the fusion index we looked for common explanation. The expression of both transcription factors MYOD and MYOG was downregulated at some point during the six-day differentiation, both by knocking out or during overexpression of the CAP1. Thus, there might be a common denominator as far as the regulation of the myogenic transcription factors is concerned. It would be important to further explore the mechanism of the regulation of the myogenic transcription factor *via* CAP1 perturbation.

We identified miRNA-mediated mRNA degradation as a mechanism by which *Cap1* is regulated. Both mRNA and protein levels were reduced during differentiation. It is well established that miRNAs play an important role during the myogenic differentiation process, and we and others have previously validated the upregulation of myomiRs whose expression increase manifold (Sjogren et al., 2017; Holstein et al., 2020; Singh et al., 2020; Xu et al., 2020). We searched for the candidate miRNAs with potential binding sites at the 3′-UTR of *Cap1*, using our own published set of myomiRs in C2C12 cells (GSE136956) (Holstein et al., 2020). Transient transfection of mir-1, mir-133 and miR-206 downregulates the expression of *Cap1* both at mRNA and protein level, in contrast to the myomiRs mir-378 and miR-486 lacking an obvious 3′-UTR binding site, although, expression of all the tested miRNAs were shown to increase during myognesis (Figure 5A). Further, 3′-UTR sequence alignment from mouse, human, chicken, dog and rat show the conserved binding sites for the seed sequence of the tested miRNAs, indicating the conserved nature of the mechanism of *Cap1* regulation (Supplementary Figure S8). Endogenous 3′-UTR deletion inhibited the downregulation of CAP1 and consequently impaired C2C12 differentiation, similar to the effects observed upon ectopic CAP1 expression (Figure 6). At this point, however, other potential mechanisms of *Cap1* regulation cannot be ruled out.

**FIGURE 7:**
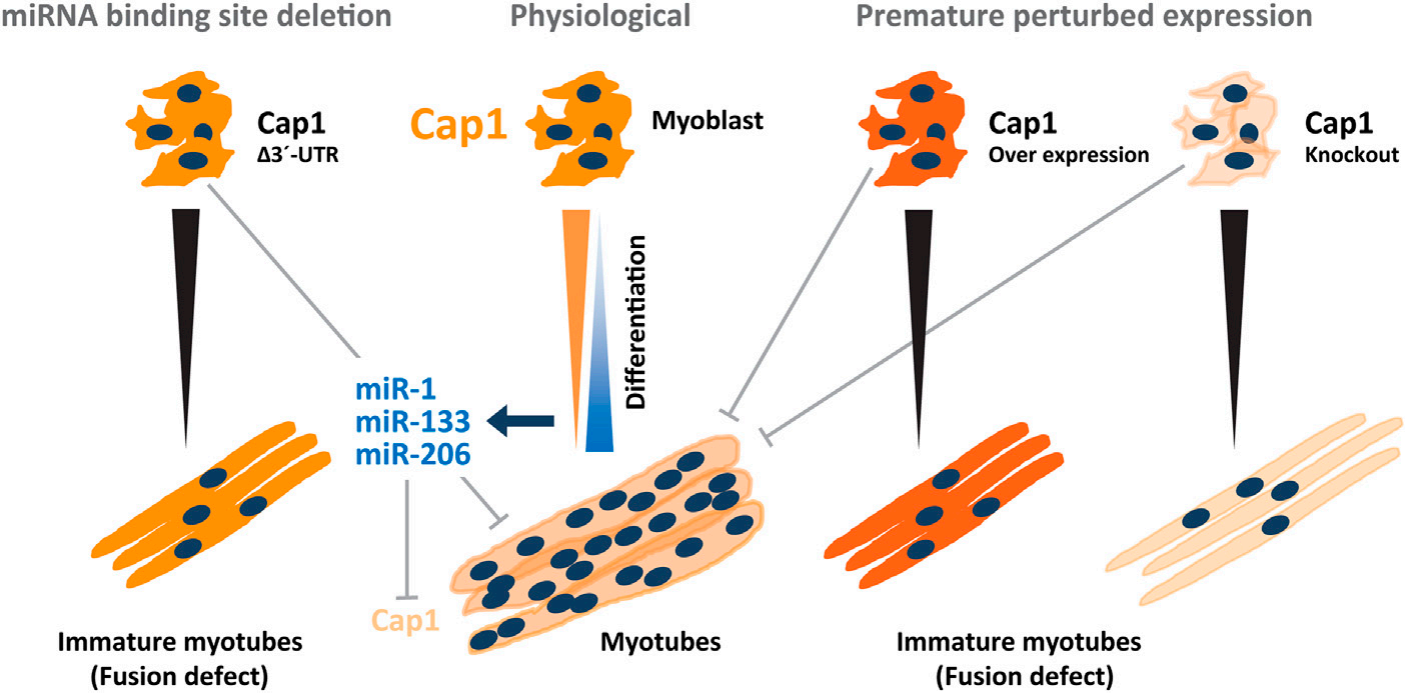
Model depicting the regulatory circuitry of myogenic C2C12 differentiation *via* post-transcriptional *Cap1* regulation. Under physiological conditions, a timely and necessary downregulation of the *Cap1* during myogenesis, is induced by myogenic miRNAs miR-1a-3p, miR-133a-3p, and miR-206-3p whose expression increases manifold during differentiation. The decreased levels of CAP1 increases the F-actin levels that enable myoblasts for elongation, migration and fusion necessary for the myoblasts fusion and myotube maturation. Under experimental conditions, both at the induced overexpression and knockout scenario (on the right side) a decreased fusion index was observed as measured by the thickness of the myotubes as well as the number of the nuclei present in the myosin heavy chain positive myotubes. Endogenous deletion of the *Cap1* 3′ UTR (on the left side) also resulted in the diminished fusion index similar to the CAP1 overexpressing myoblast. Overall, a timely decrease in the expression of the CAP1 is necessary for myoblast fusion.

Taken together, *Cap1* expression occurs in murine and human myoblasts and has an important role during myogenic differentiation. *In vivo*, muscle injury may activate quiescent stem cells such as satellite cells to form proliferating myoblasts, which profoundly express CAP1 protein. Moreover, timely downregulation of *Cap1* by miRNA-mediated posttranscriptional control is required for upregulation of F-actin and further for the fusion process (Figure 7). Such a model is consistent with previous findings that CAP1 is expressed at the embryonic stage and upon injury, while its expression subsides upon healing and in the adult. We speculate that at later stages of the differentiation, CAP2 and probably other actin modulators compensate for CAP1 loss in myotubes. The mechanistic details and functional differences of these temporal changes, however, remain to be investigated.

## Data Availability

The original contributions presented in the study are included in the article/Supplementary Material, further inquiries can be directed to the corresponding authors.

## References

[B1] Abmayr S. M. Pavlath G. K. (2012). Myoblast fusion: Lessons from flies and mice. Development 139 (4), 641–656. 10.1242/dev.068353 22274696 PMC3265056

[B2] Aspit L. Levitas A. Etzion S. Krymko H. Slanovic L. Zarivach R. (2019). CAP2 mutation leads to impaired actin dynamics and associates with supraventricular tachycardia and dilated cardiomyopathy. J. Med. Genet. 56 (4), 228–235. 10.1136/jmedgenet-2018-105498 30518548

[B3] Bertling E. Hotulainen P. Mattila P. K. Matilainen T. Salminen M. Lappalainen P. (2004). Cyclase-associated protein 1 (CAP1) promotes cofilin-induced actin dynamics in mammalian nonmuscle cells. Mol. Biol. Cell 15 (5), 2324–2334. 10.1091/mbc.e04-01-0048 15004221 PMC404026

[B4] Biederer C. H. Ries S. J. Moser M. Florio M. Israel M. A. McCormick F. (2000). The basic helix-loop-helix transcription factors myogenin and Id2 mediate specific induction of caveolin-3 gene expression during embryonic development. J. Biol. Chem. 275 (34), 26245–26251. 10.1074/jbc.M001430200 10835421

[B5] Chen I. H. Huber M. Guan T. Bubeck A. Gerace L. (2006). Nuclear envelope transmembrane proteins (NETs) that are up-regulated during myogenesis. BMC Cell Biol. 7, 38. 10.1186/1471-2121-7-38 17062158 PMC1635557

[B6] Demonbreun A. R. Biersmith B. H. McNally E. M. (2015). Membrane fusion in muscle development and repair. Semin. Cell Dev. Biol. 45, 48–56. 10.1016/j.semcdb.2015.10.026 26537430 PMC4679555

[B7] Deng S. Bothe I. Baylies M. K. (2015). The formin diaphanous regulates myoblast fusion through actin polymerization and arp2/3 regulation. PLoS Genet. 11 (8), e1005381. 10.1371/journal.pgen.1005381 26295716 PMC4546610

[B8] Duan R. Gallagher P. J. (2009). Dependence of myoblast fusion on a cortical actin wall and nonmuscle myosin IIA. Dev. Biol. 325 (2), 374–385. 10.1016/j.ydbio.2008.10.035 19027000 PMC2823627

[B9] Edgar R. Domrachev M. Lash A. E. (2002). Gene expression omnibus: NCBI gene expression and hybridization array data repository. Nucleic Acids Res. 30 (1), 207–210. 10.1093/nar/30.1.207 11752295 PMC99122

[B10] Field J. Ye D. Z. Shinde M. Liu F. Schillinger K. J. Lu M. (2015). CAP2 in cardiac conduction, sudden cardiac death and eye development. Sci. Rep. 5, 17256. 10.1038/srep17256 26616005 PMC4663486

[B11] Gurniak C. B. Chevessier F. Jokwitz M. Jönsson F. Perlas E. Richter H. (2014). Severe protein aggregate myopathy in a knockout mouse model points to an essential role of cofilin2 in sarcomeric actin exchange and muscle maintenance. Eur. J. Cell Biol. 93 (5-6), 252–266. 10.1016/j.ejcb.2014.01.007 24598388

[B12] Haralalka S. Shelton C. Cartwright H. N. Katzfey E. Janzen E. Abmayr S. M. (2011). Asymmetric Mbc, active Rac1 and F-actin foci in the fusion-competent myoblasts during myoblast fusion in Drosophila. Development 138 (8), 1551–1562. 10.1242/dev.057653 21389053 PMC3062424

[B13] Holstein I. Singh A. K. Pohl F. Misiak D. Braun J. Leitner L. (2020). Post-transcriptional regulation of MRTF-A by miRNAs during myogenic differentiation of myoblasts. Nucleic Acids Res. 48 (16), 8927–8942. 10.1093/nar/gkaa596 32692361 PMC7498330

[B14] Hubberstey A. V. Mottillo E. P. (2002). Cyclase-associated proteins: CAPacity for linking signal transduction and actin polymerization. FASEB J. 16 (6), 487–499. 10.1096/fj.01-0659rev 11919151

[B15] Huijbregts J. White J. D. Grounds M. D. (2001). The absence of MyoD in regenerating skeletal muscle affects the expression pattern of basement membrane, interstitial matrix and integrin molecules that is consistent with delayed myotube formation. Acta Histochem. 103 (4), 379–396. 10.1078/0065-1281-00607 11700944

[B16] Jang H. D. Lee S. E. Yang J. Lee H. C. Shin D. Lee H. (2020). Cyclase-associated protein 1 is a binding partner of proprotein convertase subtilisin/kexin type-9 and is required for the degradation of low-density lipoprotein receptors by proprotein convertase subtilisin/kexin type-9. Eur. Heart J. 41 (2), 239–252. 10.1093/eurheartj/ehz566 31419281 PMC6945527

[B17] Johnston A. B. Collins A. Goode B. L. (2015). High-speed depolymerization at actin filament ends jointly catalysed by Twinfilin and Srv2/CAP. Nat. Cell Biol. 17 (11), 1504–1511. 10.1038/ncb3252 26458246 PMC4808055

[B18] Kepser L. J. Damar F. De Cicco T. Chaponnier C. Prószyński T. J. Pagenstecher A. (2019). CAP2 deficiency delays myofibril actin cytoskeleton differentiation and disturbs skeletal muscle architecture and function. Proc. Natl. Acad. Sci. U. S. A. 116 (17), 8397–8402. 10.1073/pnas.1813351116 30962377 PMC6486752

[B19] Kim S. Shilagardi K. Zhang S. Hong S. N. Sens K. L. Bo J. (2007). A critical function for the actin cytoskeleton in targeted exocytosis of prefusion vesicles during myoblast fusion. Dev. Cell 12 (4), 571–586. 10.1016/j.devcel.2007.02.019 17419995

[B20] Kotila T. Wioland H. Enkavi G. Kogan K. Vattulainen I. Jegou A. (2019). Mechanism of synergistic actin filament pointed end depolymerization by cyclase-associated protein and cofilin. Nat. Commun. 10 (1), 5320. 10.1038/s41467-019-13213-2 31757941 PMC6876575

[B21] Labun K. Montague T. G. Krause M. Torres Cleuren Y. N. Tjeldnes H. Valen E. (2019). CHOPCHOP v3: Expanding the CRISPR web toolbox beyond genome editing. Nucleic Acids Res. 47 (W1), W171–w174. 10.1093/nar/gkz365 31106371 PMC6602426

[B22] Lukjanenko L. Brachat S. Pierrel E. Lach-Trifilieff E. Feige J. N. (2013). Genomic profiling reveals that transient adipogenic activation is a hallmark of mouse models of skeletal muscle regeneration. PLoS One 8 (8), e71084. 10.1371/journal.pone.0071084 23976982 PMC3744575

[B23] Luo W. Li E. Nie Q. Zhang X. (2015). Myomaker, regulated by MYOD, MYOG and miR-140-3p, promotes chicken myoblast fusion. Int. J. Mol. Sci. 16 (11), 26186–26201. 10.3390/ijms161125946 26540045 PMC4661805

[B24] Luo W. Lin Z. Chen J. Chen G. Zhang S. Liu M. (2021). TMEM182 interacts with integrin beta 1 and regulates myoblast differentiation and muscle regeneration. J. Cachexia Sarcopenia Muscle 12 (6), 1704–1723. 10.1002/jcsm.12767 34427057 PMC8718073

[B25] Mu A. Fung T. S. Francomacaro L. M. Huynh T. Kotila T. Svindrych Z. (2020). Regulation of INF2-mediated actin polymerization through site-specific lysine acetylation of actin itself. Proc. Natl. Acad. Sci. U. S. A. 117 (1), 439–447. 10.1073/pnas.1914072117 31871199 PMC6955303

[B26] Nowak S. J. Nahirney P. C. Hadjantonakis A. K. Baylies M. K. (2009). Nap1-mediated actin remodeling is essential for mammalian myoblast fusion. J. Cell Sci. 122 (18), 3282–3293. 10.1242/jcs.047597 19706686 PMC2736864

[B27] Ohtake Y. Tojo H. Seiki M. (2006). Multifunctional roles of MT1-MMP in myofiber formation and morphostatic maintenance of skeletal muscle. J. Cell Sci. 119 (18), 3822–3832. 10.1242/jcs.03158 16926191

[B28] Ono S. (2013). The role of cyclase-associated protein in regulating actin filament dynamics - more than a monomer-sequestration factor. J. Cell Sci. 126 (15), 3249–3258. 10.1242/jcs.128231 23908377 PMC3730240

[B29] Peche V. Shekar S. Leichter M. Korte H. Schroder R. Schleicher M. (2007). CAP2, cyclase-associated protein 2, is a dual compartment protein. Cell. Mol. Life Sci. 64 (19-20), 2702–2715. 10.1007/s00018-007-7316-3 17805484 PMC11135998

[B30] Peche V. S. Holak T. A. Burgute B. D. Kosmas K. Kale S. P. Wunderlich F. T. (2013). Ablation of cyclase-associated protein 2 (CAP2) leads to cardiomyopathy. Cell. Mol. Life Sci. 70 (3), 527–543. 10.1007/s00018-012-1142-y 22945801 PMC11113306

[B31] Peckham M. (2008). Engineering a multi-nucleated myotube, the role of the actin cytoskeleton. J. Microsc. 231 (3), 486–493. 10.1111/j.1365-2818.2008.02061.x 18755004

[B32] Pelucchi S. Vandermeulen L. Pizzamiglio L. Aksan B. Yan J. Konietzny A. (2020). Cyclase-associated protein 2 dimerization regulates cofilin in synaptic plasticity and Alzheimer's disease. Brain Commun. 2 (2), fcaa086. 10.1093/braincomms/fcaa086 33094279 PMC7566557

[B33] Pfaffl M. W. (2001). A new mathematical model for relative quantification in real-time RT-PCR. Nucleic Acids Res. 29 (9), e45. 10.1093/nar/29.9.e45 11328886 PMC55695

[B34] Richardson B. E. Beckett K. Nowak S. J. Baylies M. K. (2007). SCAR/WAVE and Arp2/3 are crucial for cytoskeletal remodeling at the site of myoblast fusion. Development 134 (24), 4357–4367. 10.1242/dev.010678 18003739 PMC2880884

[B35] Rochlin K. Yu S. Roy S. Baylies M. K. (2010). Myoblast fusion: When it takes more to make one. Dev. Biol. 341 (1), 66–83. 10.1016/j.ydbio.2009.10.024 19932206 PMC2854170

[B36] Rust M. B. Khudayberdiev S. Pelucchi S. Marcello E. (2020). CAPt’n of actin dynamics: Recent advances in the molecular, developmental and physiological functions of cyclase-associated protein (CAP). Front. Cell Dev. Biol. 8 (961), 586631. 10.3389/fcell.2020.586631 33072768 PMC7543520

[B37] Sanjana N. E. Shalem O. Zhang F. (2014). Improved vectors and genome-wide libraries for CRISPR screening. Nat. Methods 11 (8), 783–784. 10.1038/nmeth.3047 25075903 PMC4486245

[B38] Schneider F. Duong T. A. Metz I. Winkelmeier J. Hübner C. A. Endesfelder U. (2021). Mutual functional dependence of cyclase-associated protein 1 (CAP1) and cofilin1 in neuronal actin dynamics and growth cone function. Prog. Neurobiol. 202, 102050. 10.1016/j.pneurobio.2021.102050 33845164

[B39] Sens K. L. Zhang S. Jin P. Duan R. Zhang G. Luo F. (2010). An invasive podosome-like structure promotes fusion pore formation during myoblast fusion. J. Cell Biol. 191 (5), 1013–1027. 10.1083/jcb.201006006 21098115 PMC2995175

[B40] Shekhar S. Chung J. Kondev J. Gelles J. Goode B. L. (2019). Synergy between Cyclase-associated protein and Cofilin accelerates actin filament depolymerization by two orders of magnitude. Nat. Commun. 10 (1), 5319. 10.1038/s41467-019-13268-1 31757952 PMC6876572

[B41] Singh G. B. Cowan D. B. Wang D. Z. (2020). Tiny regulators of massive tissue: MicroRNAs in skeletal muscle development, myopathies, and cancer cachexia. Front. Oncol. 10, 598964. 10.3389/fonc.2020.598964 33330096 PMC7719840

[B42] Sjogren R. J. O. Lindgren Niss M. H. L. Krook A. (2017). “Skeletal muscle microRNAs: Roles in differentiation, disease and exercise,” in Hormones, metabolism and the benefits of exercise. Editor Spiegelman B. (Cham: Springer), 67–81. 31314463

[B43] Stadler B. Blattler T. M. Franco-Obregon A. (2010). Time-lapse imaging of *in vitro* myogenesis using atomic force microscopy. J. Microsc. 237 (1), 63–69. 10.1111/j.1365-2818.2009.03302.x 20055919

[B44] Stöckigt F. Peche V. S. Linhart M. Nickenig G. Noegel A. A. Schrickel J. W. (2016). Deficiency of cyclase-associated protein 2 promotes arrhythmias associated with connexin43 maldistribution and fibrosis. Arch. Med. Sci. 12 (1), 188–198. 10.5114/aoms.2015.54146 26925136 PMC4754362

[B45] Swailes N. T. Knight P. J. Peckham M. (2004). Actin filament organization in aligned prefusion myoblasts. J. Anat. 205 (5), 381–391. 10.1111/j.0021-8782.2004.00341.x 15575887 PMC1571354

[B46] Vasyutina E. Martarelli B. Brakebusch C. Wende H. Birchmeier C. (2009). The small G-proteins Rac1 and Cdc42 are essential for myoblast fusion in the mouse. Proc. Natl. Acad. Sci. U. S. A. 106 (22), 8935–8940. 10.1073/pnas.0902501106 19443691 PMC2682539

[B47] Werner S. Lützkendorf J. Müller T. Müller L. P. Posern G. (2019). MRTF-A controls myofibroblastic differentiation of human multipotent stromal cells and their tumour-supporting function in xenograft models. Sci. Rep. 9 (1), 11725. 10.1038/s41598-019-48142-z 31409840 PMC6692381

[B48] Xu M. Chen X. Chen D. Yu B. Li M. He J. (2020). Regulation of skeletal myogenesis by microRNAs. J. Cell. Physiol. 235 (1), 87–104. 10.1002/jcp.28986 31230374

[B49] Zhang H. Ghai P. Wu H. Wang C. Field J. Zhou G. L. (2013). Mammalian adenylyl cyclase-associated protein 1 (CAP1) regulates cofilin function, the actin cytoskeleton, and cell adhesion. J. Biol. Chem. 288 (29), 20966–20977. 10.1074/jbc.M113.484535 23737525 PMC3774366

[B50] Zhang H. Wen J. Bigot A. Chen J. Shang R. Mouly V. (2020). Human myotube formation is determined by MyoD-Myomixer/Myomaker axis. Sci. Adv. 6 (51), eabc4062. 10.1126/sciadv.abc4062 33355126 PMC11206528

