# Supplementary material for: miRNA mediated downregulation of cyclase-associated protein 1 (CAP1) is required for myoblast fusion
Source: Front Cell Dev Biol. 2022 Sep 30;10:899917. doi: 10.3389/fcell.2022.899917 (PMC9562714; doi:10.3389/fcell.2022.899917)
Supplement: Supplementary file 1 [file Table1.docx]

Supplementary Material

# Supplementary Figures and Tables

## Supplementary Figures


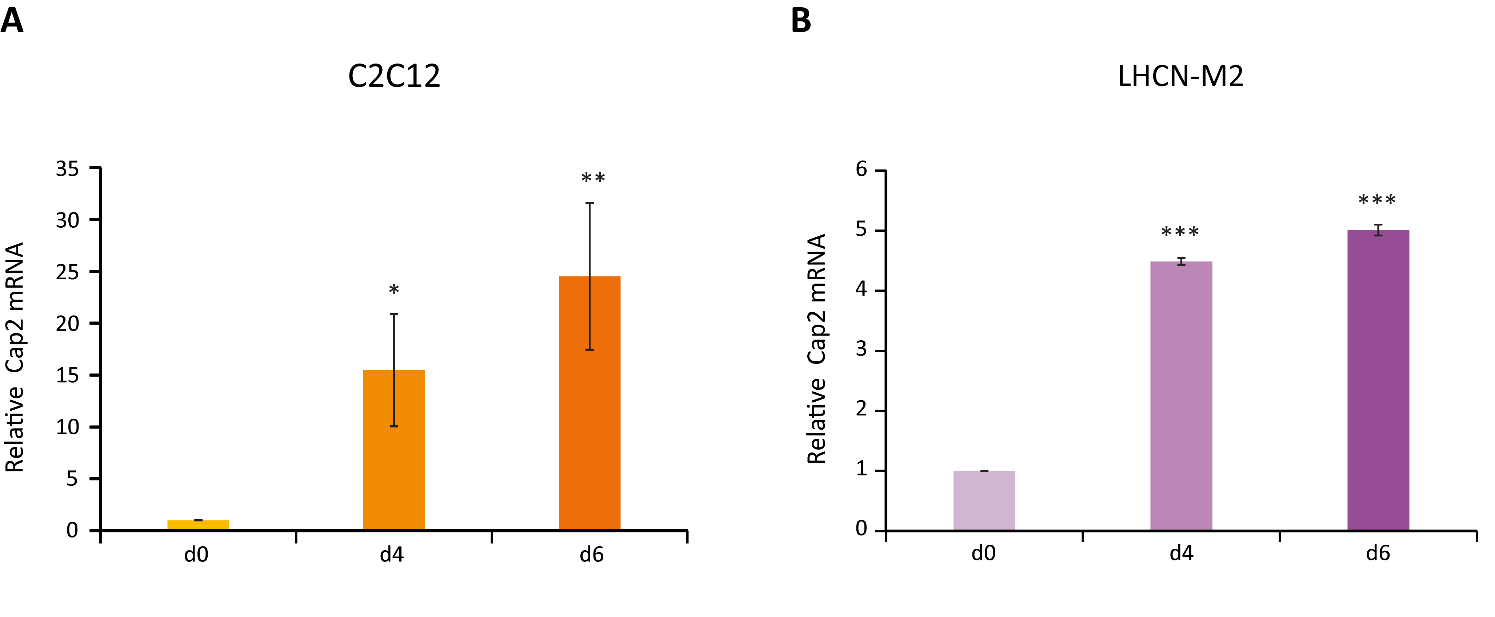


**Fig. S1** *Cap2* mRNA increases during myogenic differentiation of the murine and human myoblast cell lines. (**A**) Expression of the *Cap2* increases significantly in the C2C12 cells during differentiation day 4 and day 6 (d4 and d6) as compared to the undifferentiated control cells (d0). (**B**) A similar increase in the *Cap2* mRNA level was observed in the case of human myoblast cell line LHCN-M2. Error bars, SEM (n=3); *p< 0.05, **p< 0.01, ***p< 0.001 (Student’s t-test).

**
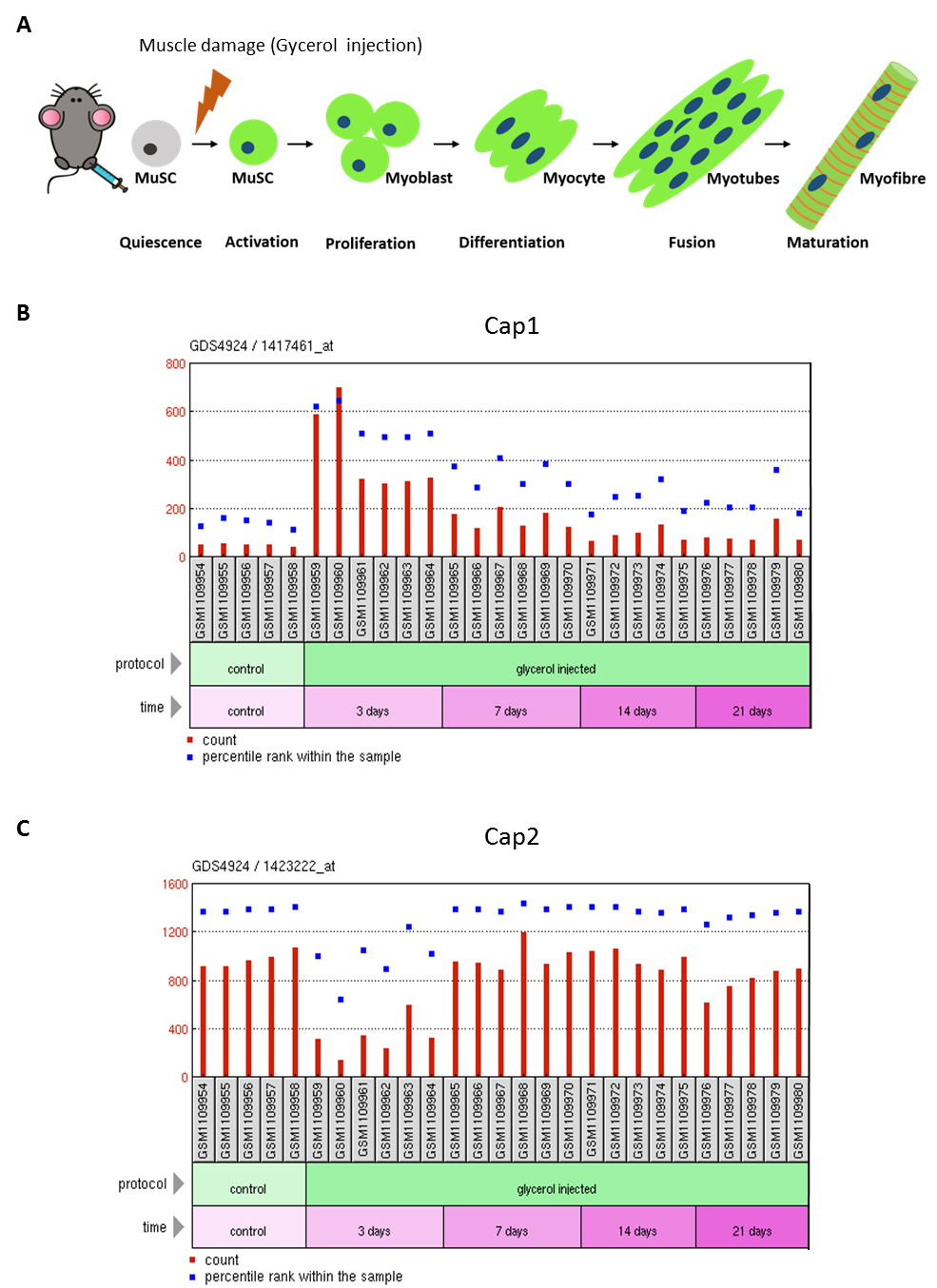
**

**Fig. S2** Re-analysis of GEO dataset GSE45577, showing induction of *Cap1* expression after injury of skeletal muscle *in vivo*. (**A**) Schematics of the experimental setup for the mouse skeletal muscle injury model by (Lukjanenko et al., 2013). (**B**) Expression of *Cap1* in the *Tibialis anterior* muscle of 12 week old C57BL/6J males before and after injection of glycerol, using the Affymetrix Mouse Genome 430 2.0 Array. Significant upregulation/re-expression of *Cap1* occurs 3 days post-injury, followed by subsequent downregulation towards 21 days when regenerated mature myotubes show only diminished *Cap1* expression (<https://www.ncbi.nlm.nih.gov/geoprofiles/113963492>). (**C**) *Cap2* expression showing inverse regulation upon regeneration, with low-level expression in muscle 3 days after injury (<https://www.ncbi.nlm.nih.gov/geoprofiles/113969228>).

**
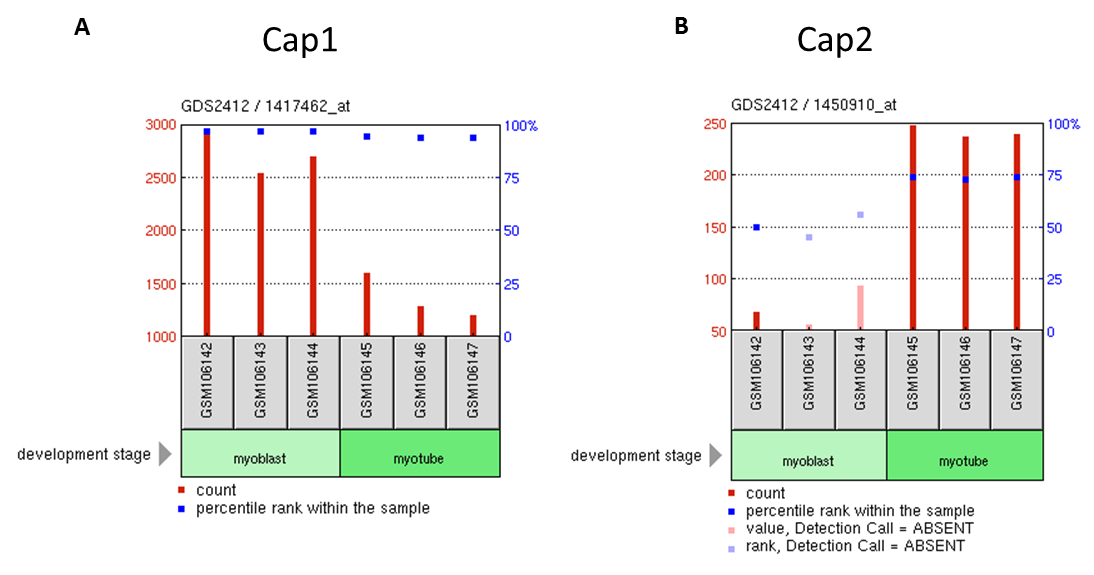
**

**Fig. S3** Re-analysis of GEO dataset GSE4694, showing diminished *Cap1* expression upon myogenic differentiation of C2C12 cells. (**A**) Expression of *Cap1* in the Affymetrix Mouse Genome 430 2.0 microarray by (Chen et al., 2006). Compared to the myoblast population of C2C12 cells, differentiated myotubes show diminished expression of *Cap1* (<https://www.ncbi.nlm.nih.gov/geoprofiles/31829693>). (**B**) Inverse regulation of *Cap2*, being almost undetectable in undifferentiated C2C12 myoblasts (<https://www.ncbi.nlm.nih.gov/geoprofiles/31863105>).





**Fig. S4** CRISPR-Cas9 mediated-generation of *Cap1* knockout in C2C12 cells. Agarose gel picture of PCR conducted on the genomic DNA isolated from Cas9 control and cells transduced with two sg-RNA against *Cap1*. Different DNA polymerase were used as mentioned below the respective lanes. Taq DNA polymerase shows an amplicon of around 1.3 Kb in the sg-RNA treated cells corresponding to the intended deletion. Long-Amp polymerase shows an amplicon of around 7 Kb corresponding to the calculated size flanking the two guide RNA and a band of around 1.3 Kb in case of the sg-RNA treated cells, corresponding to deletion of *Cap1* by both the guide RNA.


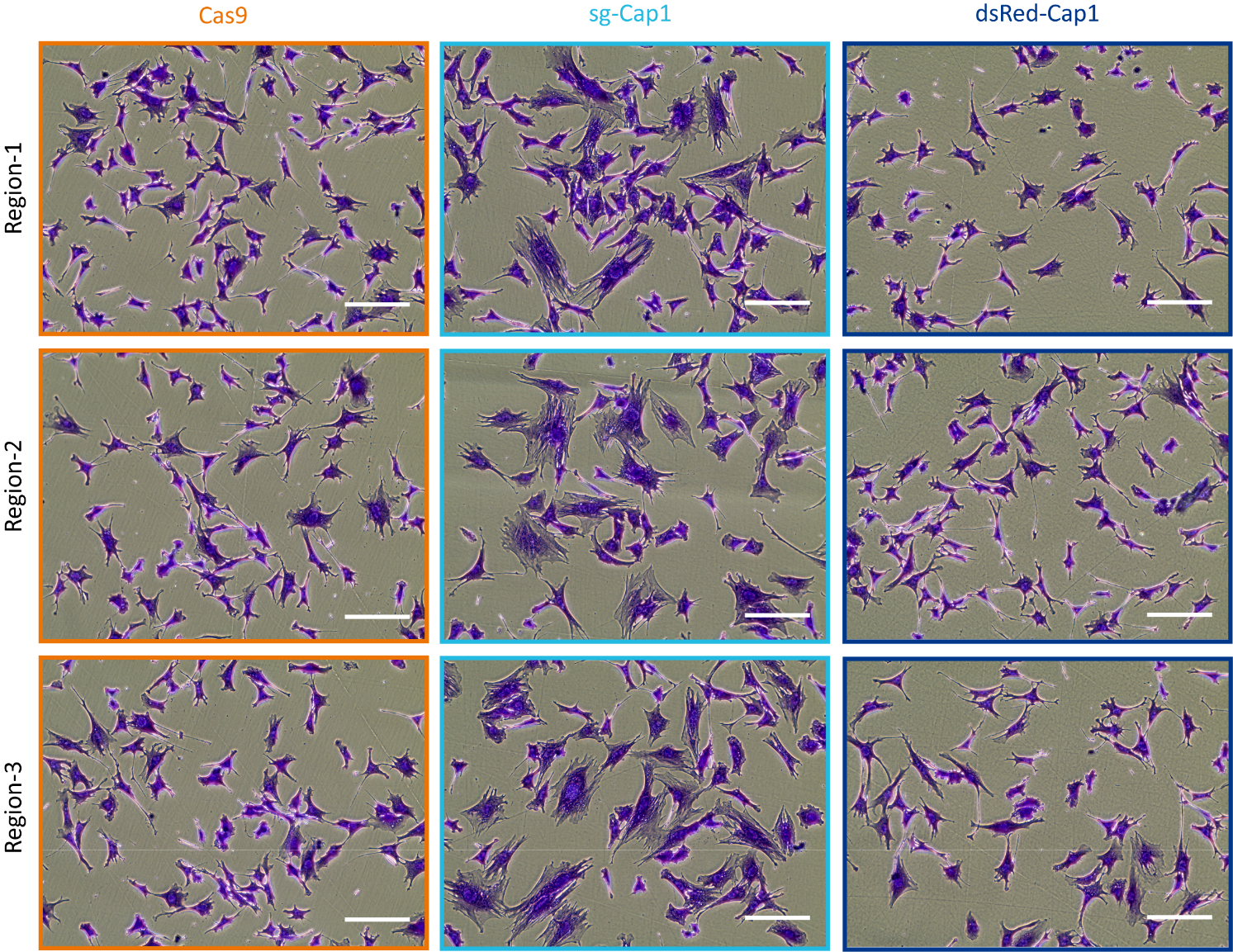


**Fig. S5** Knockout (KO) of *Cap1* resulted in increased cell size. Crystal violet staining of the CRISPR-Cas9 mediated *Cap1* KO (sg-Cap1; mixed population, middle panel), shows a much bigger cell size as compared to the Cas9 control cells (left panel). While overexpression (OE) of the tagged *Cap1* (dsRed-Cap1) resulted in the decreased cell size (right panel). Microscopic pictures from three different areas are presented in the figure. Scale bar, 200 μm.

**
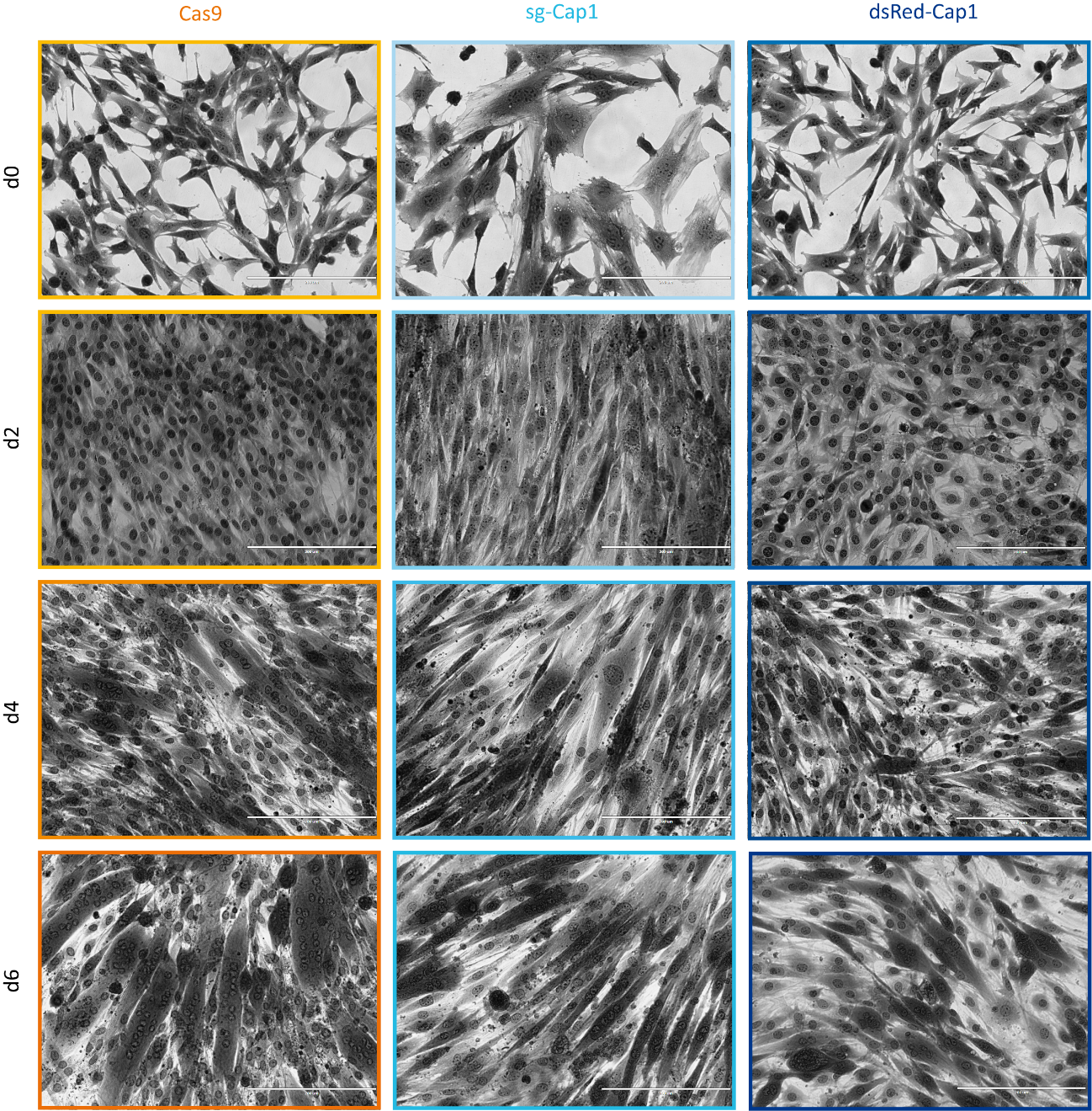
**

**Fig. S6** Perturbation in CAP1 expression affects the myogenic differentiation of C2C12 cells. Bright-field images (20x) of crystal violet stained C2C12 cells upon differentiation for 2, 4 and 6 days (d2, d4, d6), in comparison to undifferentiated control cells (d0). On day2 (d2) of the differentiation, both the Cas9 controls as well as the KO cells (sg-Cap1) show a more elongated phenotype, while CAP1 OE cells (dsRed-Cap1), still showed more fibroblast (parental) phenotype. Day4 (d4), of the differentiation, shows the appearance of multinucleated myotubes in the case of the Cas9 cells, while in case of the KO as well as OE cells they appear to have a single nucleus per cell. CAP1 over-expressing cells show now elongated phenotype on the day4 of the differentiation. On day6 (d6) of the differentiation Cas9 cells showed more mature myotubes with more numbers of nuclei per myotube as compared to both KO and OE cells line, which also showed the multinucleated myotubes but the number of nuclei per myotubes was significantly low. Scale bar, 200 μm.


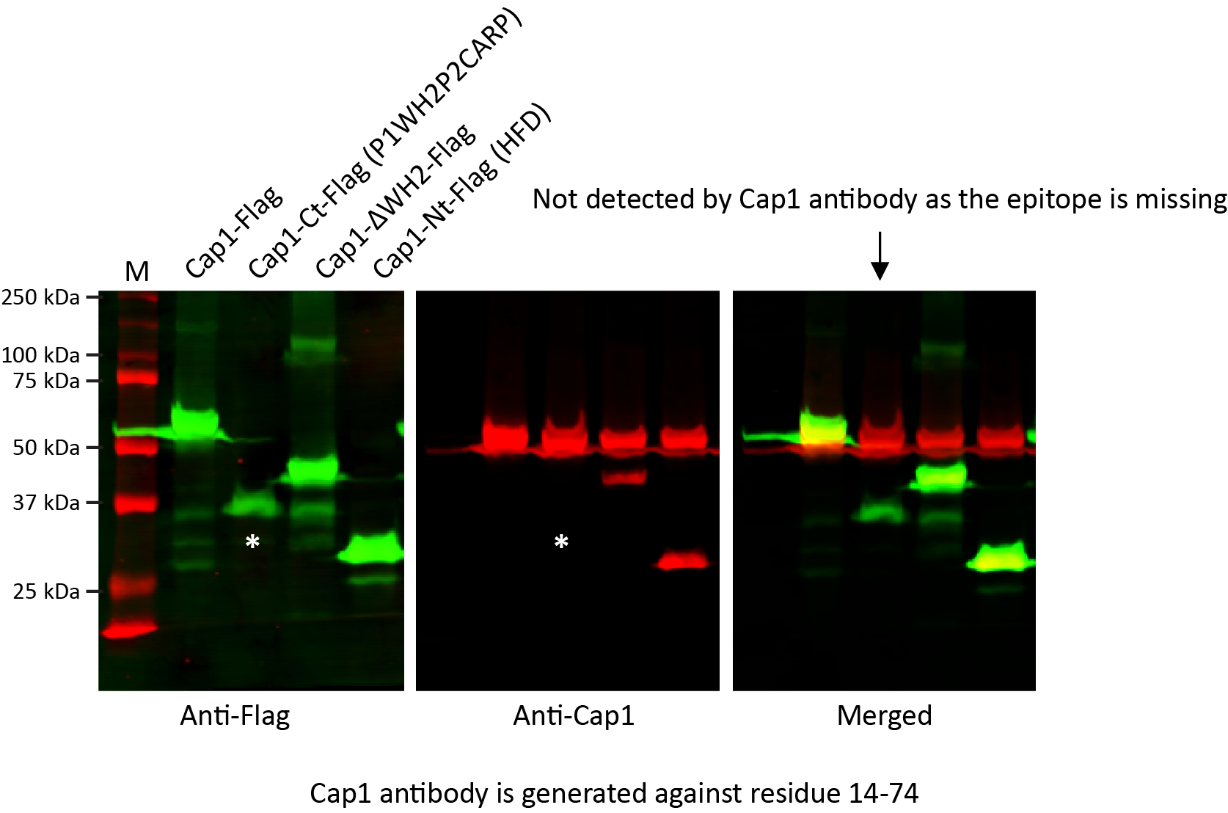


**Fig. S7** Characterization of CAP1 antibody. Western blot analyses of different *Cap1* mutant overexpressed in the HEK293T cells and detected either by Flag (green) or Cap1 (red) antibody. The Flag-antibody could detect all the mutants, while CAP1 antibody failed to detect the mutant where the epitope was missing against which the CAP1 antibody was generated as shown by asterisk. CAP1 antibody also detected the endogenous expression of the CAP1 (~ 52 kDa; red) in HEK293T cells.

**
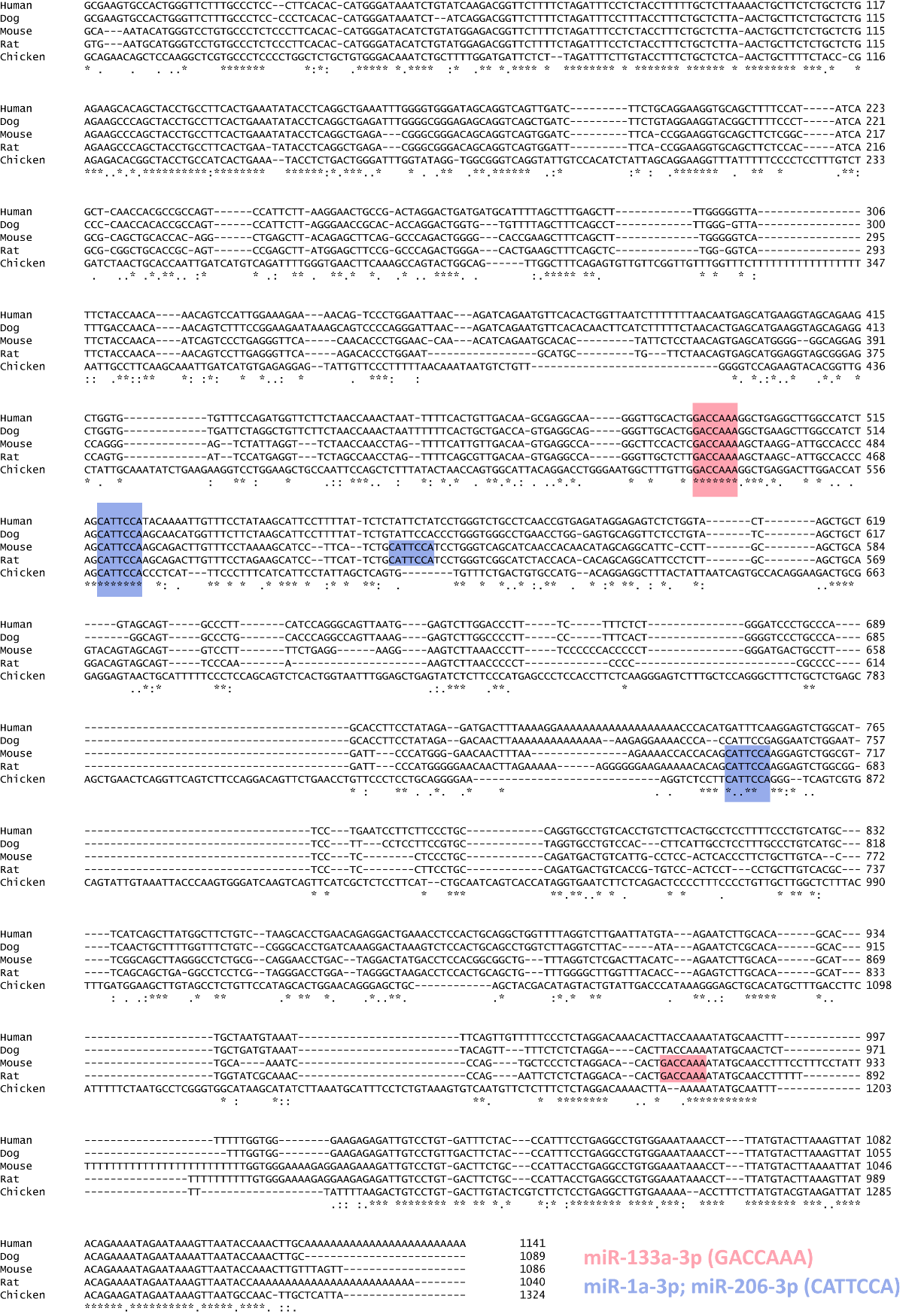
**

**Fig. S8** miRNA binding sites are conserved among different species. Multiple sequence alignment (Madeira et al., 2019) of *Cap1* 3’ UTR from different organisms shows conserved seed sequences for the tested miRNA. Red boxes indicate the seed sequence for the binding site for miR-133a-3p, and blue boxes for the miR-1a-3p and miR-206-3p, which share the same seed sequences.

**
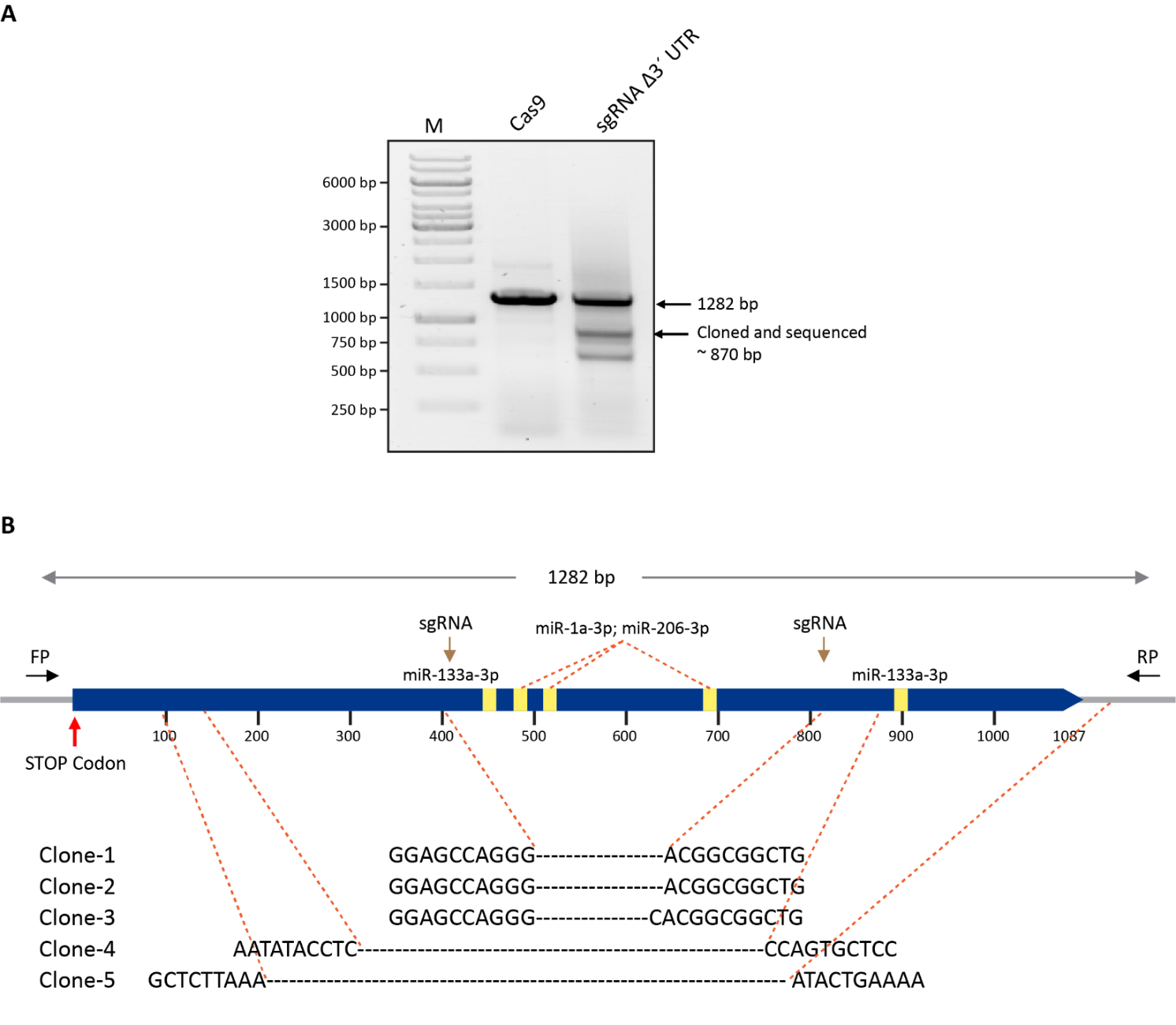
**

**Fig. S9** CRISPR-Cas9 mediated deletion of 3’-UTR of *Cap1* endogenous locus. (**A**) DNA-Agarose gel from the PCR primers depicted in the schematic diagram (**B**). The appearance of the smaller fragment represents the cell population with the deleted part of the 3’-UTR at the endogenous locus (mixed population). The smaller PCR amplicon (~870 bp) from the gel was cloned and sent for sequencing to confirm the deletion. (**B**) Schematic of the 3′-UTR of murine *Cap1* with miRNA binding sites indicated by yellow boxes and sgRNA target sites indicated by an arrow and forward and reverse primer binding position corresponding to the genomic DNA. Different clones show corresponding deleted region at the genomic locus (dotted lines).

## Supplementary Table S1: Primer list used for the generation and detection of CRISPR/Cas9 KO cells and quantitative real-time PCR.

| **Primer** | **Sequence** | **Description** |
| --- | --- | --- |
| ALAS1-F (human) | CTGCAAAGATCTGACCCCTC | qRT-PCR |
| ALAS1-R (human) | CCTCATCCACGAAGGTGATT | qRT-PCR |
| GAPDH-F (human) | ACCCAGAAGACTGTGGATGG | qRT-PCR |
| GAPDH-R (human) | TTCTAGACGGCAGGTCAGGT | qRT-PCR |
| CAP1-F (human) | AGGTGAGGCGGAACTCTGA | qRT-PCR |
| CAP1-R (human) | TGCTCCTGCTTTTGAAGGACT | qRT-PCR |
| CAP2-F (huamn) | CTCCGTGGAAGCCTTTGACA | qRT-PCR |
| CAP2-R (human) | CGTCATTCTCGTGGGGTTGT | qRT-PCR |
|  |  |  |
| HPRT-F (murine) | AAGCTTGCTGGTGAAAAGGA | qRT-PCR |
| HPRT-R (murine) | TTGCGCTCATCTTAGGCTTT | qRT-PCR |
| GAPDH-F (murine) | GGTATCGTGGAAGGACTCATG | qRT-PCR |
| GAPDH-R (murine) | ATGCCAGTGAGCTTCCCGTTC | qRT-PCR |
| CAP1-F (murine) | CTTCCGGGAGAAGAACCGAG | qRT-PCR |
| CAP1-R (murine) | TGACCCAGTCCACATGCTTC | qRT-PCR |
| CAP2-F (murine) | CGCGCCCGTCTGTATTTG | qRT-PCR |
| CAP2-R (murine) | GTCCCGCCATGTCTGTCATT | qRT-PCR |
| ß1D-Integrin-F (murine) | CATCCCAATTGTAGCAGGCG | qRT-PCR |
| ß1D-Integrin-R (murine) | GAGACCAGCTTTACGTCCATAG | qRT-PCR |
| Caveolin-3-F (murine) | GACCCCAAGAACATCAATGAGGAC | qRT-PCR |
| Caveolin-3-R (murine) | AGAAGGAGATACAGGCGAACAGGA | qRT-PCR |
| Myomaker-F (murine) | CCTGCTGTCTCTCCCAAG | qRT-PCR |
| Myomaker-R (murine) | AGAACCAGTGGGTCCCTAA | qRT-PCR |
| Myomixer-F (murine) | GTTAGAACTGGTGAGCAGGAG | qRT-PCR |
| Myomixer-R (murine) | CCATCGGGAGCAATGGAA | qRT-PCR |
| sgRNA (CAP1)-1 | ACCTTCCGGGAGAAGAACCG | sgRNA for generation of the CAP1 knockout C2C12 cells |
| sgRNA (CAP1)-2 | GTCCTCATTCCTACCGAAGG | sgRNA for generation of the CAP1 knockout C2C12 cells |
| CAP1.detect-F | AATGGAGGGAGGGGCTTTTC | for the detection of the Cap1 KO |
| CAP1.detect-R | AGGTAACCGGGCACACATAG | for the detection of the Cap1 KO |
| sgRNA cap1-3utr-1 | TAGGACTATGACCTCCACGG | sgRNA for the deletion of the CAP1 3'UTR in C2C12 cells |
| sgRNA cap1-3utr-2 | TTAGAACCTAATAGACTCCC | sgRNA for the deletion of the CAP1 3'UTR in C2C12 cells |
| 3'UTR-F | GCAGTTCAAGACCCTGTGGA | for the detection of 3'UTR deletion in C2C12 cells |
| 3'UTR-R | GGTTAAGGGCAGGAAGTGGG | for the detection of 3'UTR deletion in C2C12 cells |

##

# SI References

Chen, I.H., Huber, M., Guan, T., Bubeck, A., and Gerace, L. (2006). Nuclear envelope transmembrane proteins (NETs) that are up-regulated during myogenesis. *BMC Cell Biol* 7**,** 38. doi: 10.1186/1471-2121-7-38.

Lukjanenko, L., Brachat, S., Pierrel, E., Lach-Trifilieff, E., and Feige, J.N. (2013). Genomic profiling reveals that transient adipogenic activation is a hallmark of mouse models of skeletal muscle regeneration. *PLoS One* 8(8)**,** e71084. doi: 10.1371/journal.pone.0071084.

Madeira, F., Park, Y.M., Lee, J., Buso, N., Gur, T., Madhusoodanan, N., et al. (2019). The EMBL-EBI search and sequence analysis tools APIs in 2019. *Nucleic Acids Res* 47(W1)**,** W636-w641. doi: 10.1093/nar/gkz268.
